# Localized Nicardipine Release Implants for Prevention of Vasospasm After Aneurysmal Subarachnoid Hemorrhage: A Randomized Clinical Trial

**DOI:** 10.1001/jamaneurol.2024.2564

**Published:** 2024-08-19

**Authors:** Lars Wessels, Stefan Wolf, Tiziana Adage, Jörg Breitenbach, Claudius Thomé, Johannes Kerschbaumer, Martin Bendszus, Matthias Gmeiner, Andreas Gruber, Dorothee Mielke, Veit Rohde, Maria Wostrack, Bernard Meyer, Jens Gempt, Gerhard Bavinzski, Dorian Hirschmann, Peter Vajkoczy, Nils Hecht

**Affiliations:** 1 Department of Neurosurgery, Charité–Universitätsmedizin Berlin, corporate member of Freie Universität Berlin, Humboldt-Universität zu Berlin, and Berlin Institute of Health, Berlin, Germany; 2 Center for Stroke Research Berlin, Charité–Universitätsmedizin Berlin, corporate member of Freie Universität Berlin, Humboldt-Universität zu Berlin, and Berlin Institute of Health, Berlin, Germany; 3 Brain Implant Therapeutics (BIT) Pharma GmbH, Graz, Austria; 4 Department of Neurosurgery, Medizinische Universität Innsbruck, Innsbruck, Austria; 5 Department of Neuroradiology, Ruprecht-Karls Universität Heidelberg, Heidelberg, Germany; 6 Department of Neurosurgery, Johannes Kepler Universität Linz, Linz, Austria; 7 Department of Neurosurgery, Universitätsmedizin Göttingen, Göttingen, Germany; 8 Department of Neurosurgery, Universitätsklinikum Augsburg, Augsburg, Germany; 9 Department of Neurosurgery, Technische Universität München, Munich, Germany; 10 Department of Neurosurgery, Universitätsklinikum Hamburg, Hamburg, Germany; 11 Department of Neurosurgery, Medizinische Universität Wien, Vienna, Austria

## Abstract

**Importance:**

Cerebral vasospasm largely contributes to a devastating outcome after aneurysmal subarachnoid hemorrhage (aSAH), with limited therapeutic options.

**Objective:**

To investigate the safety and efficacy of localized nicardipine release implants positioned around the basal cerebral vasculature at risk for developing proximal vasospasm after aSAH.

**Design, Setting, and Participants:**

This single-masked randomized clinical trial with a 52-week follow-up was performed between April 5, 2020, and January 23, 2023, at 6 academic neurovascular centers in Germany and Austria. Consecutive patients with World Federation of Neurological Surgeons grade 3 or 4 aSAH due to a ruptured anterior circulation aneurysm requiring microsurgical aneurysm repair participated.

**Intervention:**

During aneurysm repair, patients were randomized 1:1 to intraoperatively receive 10 implants at 4 mg of nicardipine each plus standard of care (implant group) or aneurysm repair alone plus standard of care (control group).

**Main Outcome and Measures:**

The primary end point was the incidence of moderate to severe cerebral angiographic vasospasm (aVS) between days 7 and 9 after aneurysm rupture as determined by digital subtraction angiography.

**Results:**

Of 41 patients, 20 were randomized to the control group (mean [SD] age, 54.9 [9.1] years; 17 female [85%]) and 21 to the implant group (mean [SD] age, 53.6 [11.9] years; 14 female [67%]). A total of 39 patients were included in the primary efficacy analysis. In the control group, 11 of 19 patients (58%) developed moderate or severe aVS compared with 4 of 20 patients (20%) in the implant group (*P* = .02). This outcome was paralleled by a lower clinical need for vasospasm rescue therapy in the implant group (2 of 20 patients [10%]) compared with the control group (11 of 19 patients [58%]; *P* = .002). Between days 13 and 15 after aneurysm rupture, new cerebral infarcts were noted in 6 of 19 patients (32%) in the control group and in 2 of 20 patients (10%) in the implant group (*P* = .13). At 52 weeks, favorable outcomes were noted in 12 of 18 patients (67%) in the control group and 16 of 19 patients (84%) in the implant group (*P* = .27). The adverse event rate did not differ between groups.

**Conclusions and Relevance:**

These findings show that placing nicardipine release implants during microsurgical aneurysm repair can provide safe and effective prevention of moderate to severe aVS after aSAH. A phase 3 clinical trial to investigate the effect of nicardipine implants on clinical outcome may be warranted.

**Trial Registration:**

ClinicalTrials.gov Identifier: NCT04269408

## Key Points

**Question:**

Does a nicardipine release implant inserted during microsurgical aneurysm repair prevent angiographic vasospasm after aneurysmal subarachnoid hemorrhage (aSAH)?

**Findings:**

In this phase 2b randomized clinical trial in 41 adult patients with aSAH, the proportion experiencing moderate to severe angiographic vasospasm was significantly lower in those who received the nicardipine implant plus standard of care (20%) compared with those treated with standard of care alone (58%). The adverse event rate did not differ between groups.

**Meaning:**

These findings show that nicardipine implants may safely and effectively reduce angiographic vasospasm and may improve clinical outcomes after aSAH, warranting further investigation in a phase 3 trial.

## Teaser

This randomized clinical trial investigated the safety and efficacy of nicardipine release implants for preventing proximal angiographic vasospasm after aneurysmal subarachnoid hemorrhage.

## Introduction

Aneurysmal subarachnoid hemorrhage (aSAH) is associated with high morbidity and mortality as well as a substantial personal and socioeconomic burden. A large proportion of the neurologic injury following aSAH results from delayed cerebral ischemia (DCI).^1^ Up to 84% of patients experiencing DCI also experience proximal angiographic vasospasm (aVS) of the basal cerebral vasculature, which indicates a strong association between the occurrence of aVS and the development of DCI.^2^ So far, oral systemic nimodipine represents the only guideline-recommended pharmacologic therapy for the prevention of aVS-associated DCI but remains hampered by limited effectiveness and systemic adverse effects.^3^

In contrast, a more target-specific drug delivery strategy is to directly place vasoactive substances next to the basal cerebral vasculature most likely to develop aVS.^4^ For this purpose, NicaPlant (BIT Pharma) represents a good manufacturing practice (GMP)–compliant release formulation of the calcium channel blocker nicardipine, which is manufactured in a rod shape and can be placed in proximity to the basal cerebral vasculature at the time of microsurgical aneurysm repair to ensure a prolonged and highly localized drug delivery.^5^ Most recently, a phase 2a study confirmed the general feasibility of the nicardipine release implant and that 10 implants containing 4 mg nicardipine each represent the optimal dose.^6^ The aim of the current study was to determine the safety and efficacy of nicardipine release implant for moderate to severe aVS following aSAH.

## Methods

This phase 2b randomized clinical trial (RCT) was conducted in accordance with the Declaration of Helsinki^7^ and approved by the ethics committees in Munich, Germany and Innsbruck, Austria. Written informed consent was obtained from all participants or their legal representative. The analysis followed the Consolidated Standards of Reporting Trials (CONSORT) reporting guideline and was consistent with the protocol (Supplement 1) and statistical analysis plan.

The study was designed as an international, multicenter, single-masked RCT and performed in 6 academic neurovascular centers in Germany and Austria between April 5, 2020, and January 23, 2023. The study drug production adhered to current GMPs. Patients with World Federation of Neurological Surgeons grade 3 and 4 aSAH requiring surgical repair of a ruptured anterior circulation aneurysm were randomized 1:1 within 48 hours after aneurysm rupture to receive 10 nicardipine release implants (4 mg each) plus standard of care according to international treatment guidelines^8^ (implant group) or standard of care alone (control group). The implants were administered during surgery immediately after clipping the ruptured aneurysm and placed in proximity to the exposed major cerebral arteries based on a predefined implantation scheme.^6,9^

Randomization took place after clipping the aneurysm when the basal cisterns were opened, as would be required if pellets were implanted. Thus, the basal cisterns were opened to the same extent in both groups at the time of randomization. The surgical team then left the operating room, leaving the surgeon alone, and the patient was randomized and treated by the surgeon accordingly. Thus, only the surgeon and the person performing the randomization were not masked, and both were excluded from further interaction with physicians involved in the patient’s care. The primary efficacy end point was the incidence of moderate to severe aVS assessed by digital subtraction angiography between days 7 and 9 after aneurysm rupture. The primary safety end point was the occurrence of adverse events, defined as any untoward medical occurrence after randomization not necessarily caused by the treatment. Imaging end points were masked, core laboratory assessed, and evaluated by a neuroradiologist with more than 30 years of experience (M.B.) who was unaware of the group assignment. The evaluation was performed using anonymized datasets without information about the patient or the assigned treatment group.

### Statistical Analysis

The statistical analysis was performed using SAS, version 9.4 software (SAS Institute Inc). Demographic data included age, sex, race (Asian, Black, White, or unknown), location of the aneurysm, World Federation of Neurological Surgeons score, modified Fisher scale,^10^ and risk factors. Data on race were self-reported and included for the purpose of transparency since our patient population lacks diversity. The primary efficacy end point and other binary outcomes were evaluated using the Fisher exact test. A supporting ordinal logistic regression model was used to provide odds ratio estimates for the treatment effect and associated significance levels. Ordered categorical data were analyzed using the Cochran-Mantel-Haenszel test with modified ridit scores. Normally distributed interval data were analyzed using an analysis of variance model including treatment group as the fixed effect. Nonnormally distributed data were analyzed using a nonparametric Wilcoxon rank sum test or a rank-based analysis of variance model, as appropriate. All hypothesis tests were 2-sided, and a priori levels of significance were set at *P* < .05. Detailed descriptions of the trial design, pharmacologic properties of the study drug, and statistical analysis are provided in Supplement 1 and eMethods 1 and 2 in Supplement 2.

## Results

The analytic sample included 41 patients, with 20 randomized to the control group (mean [SD] age, 54.9 [9.1] years; 17 female [85%] and 3 male [15%]) and 21 to the implant group (mean [SD] age, 53.6 [11.9] years; 14 female [67%] and 7 male [33%]). The majority of the patients were of White race (38 [93%] compared with 3 patients of Asian, Black, or unknown race and ethnicity [7%]). Detailed information on patient characteristics (Table 1) and participant recruitment (Figure) are presented in eResults 1 and eTable 1 in Supplement 2.

**Table 1.** Patient Demographics

Characteristic	No. of patients (%)			*P* value
	Control group (n = 20)	Implant group (n = 21)	Total (n = 41)	
Age, mean (SD), y	54.9 (9.1)	53.6 (11.9)	54.2 (10.5)	.70
Sex				
Female	17 (85)	14 (67)	31 (76)	.28
Male	3 (15)	7 (33)	10 (24)	
Race				
Asian	0	1 (5)	1 (2)	.40
Black	1 (5)	0	1 (2)	
White	19 (95)	19 (90)	38 (93)	
Unknown	0	1 (5)	1 (2)	
Aneurysm location				
MCA	2 (10)	9 (43)	11 (27)	.01
ICA	9 (45)	2 (10)	11 (27)	
ACA	7 (35)	10 (48)	17 (41)	
Other	2 (10)	0	2 (5)	
WFNS grade				
3 (Moderate)	6 (30)	11 (52)	17 (41)	.21
4 (Severe)	14 (70)	10 (48)	24 (59)	
Modified Fisher scale grade^a^^,^^b^				
1	1 (5)	1 (5)	2 (5)	.55
2	0	2 (10)	2 (5)	
3	11 (55)	10 (50)	21 (53)	
4	8 (40)	7 (35)	15 (38)	
Risk factors				
Smoking	11 (55)	8 (38)	19 (46)	.29
Arterial hypertension	4 (20)	11 (52)	15 (37)	
Obesity (BMI >30)	1 (5)	1 (5)	2 (5)	
Type 2 diabetes	1 (5)	0	1 (2)	
Hyperlipoproteinemia	2 (10)	1 (5)	3 (7)	

Abbreviations: ACA, anterior communicating artery; BMI, body mass index (calculated as weight in kilograms divided by height in meters squared); ICA, internal carotid artery; MCA, middle cerebral artery; WFNS, World Federation of Neurological Surgeons.

^a^ One patient in the implant group underwent initial magnetic resonance imaging, whereas the remainder underwent computed tomography imaging.

^b^ Grade 1 includes focal or diffuse thin subarachnoid hemorrhage (SAH), no intraventricular hemorrhage (IVH), and a 24% incidence of symptomatic vasospasm; grade 2 includes focal or diffuse thin SAH, IVH present, and 33% incidence of symptomatic vasospasm; grade 3 includes thick SAH, no IVH, and 33% incidence of symptomatic vasospasm; and grade 4 includes thick SAH, IVH present, and 40% incidence of symptomatic vasospasm.^10^

**Figure.**
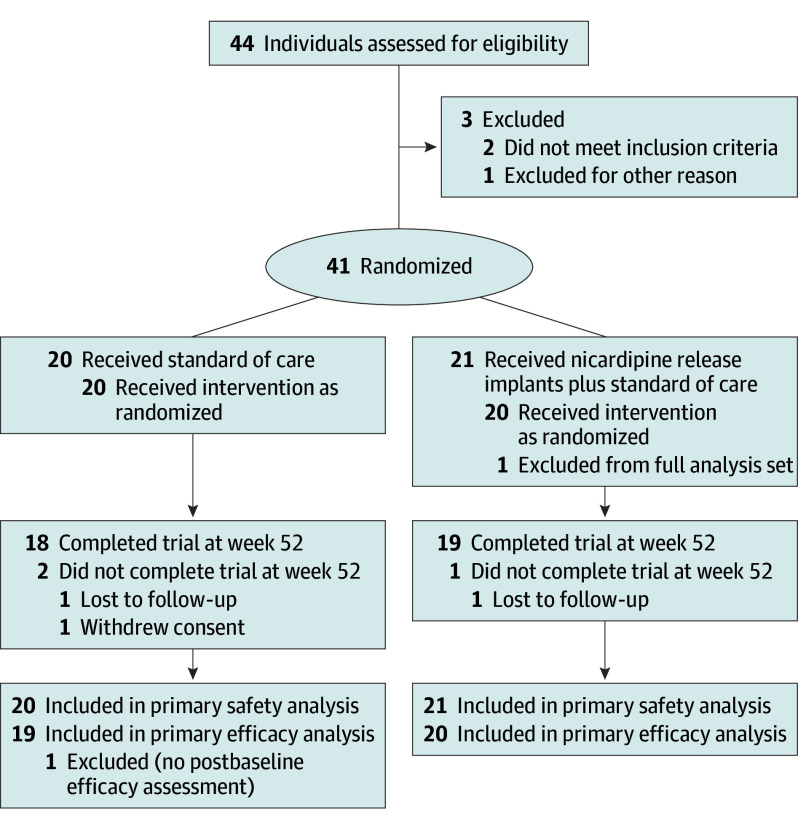
CONSORT Flow Diagram

### Primary Efficacy End Point

Between days 7 and 9 after aneurysm rupture, 11 of 19 patients (58%; 95% CI, 34%-80%) in the control group developed moderate to severe aVS compared with 4 of 20 patients (20%) (95% CI, 6%-44%) in the implant group (*P* = .02) (Table 2). The additional main proportional odds ordinal logistic regression analysis showed a significant difference between groups, with an adjusted odds ratio of 4.98 (95% CI, 1.40-17.65) indicating a high likelihood of developing any type of aVS in the control group (*P* = .01).

**Table 2.** Primary Efficacy Outcome of Angiographic Vasospasm by Severity

Treatment group	No. of patients (%)			
	None	Mild	Moderate	Severe
Control (n = 19)	5 (26)	3 (16)	6 (32)	5 (26)
Implant^a^ (n = 20)	14 (70)	2 (10)	1 (5)	3 (15)

^a^ Nicardipine implant treatment resulted in a significantly lower incidence of moderate to severe angiographic vasospasm between days 7 and 9 after aneurysm rupture (*P* = .02 for control vs implant group by Fisher exact test).

### Primary Safety End Point

Overall, the implant group showed no elevated risk profile regarding the development of treatment-emergent adverse events or serious adverse events. Until day 21, 180 treatment-emergent adverse events were reported, of which 96 occurred in the control group and 84 in the implant group. Until week 52, 16 serious adverse events were reported, of which 10 occurred in 9 of 20 patients (45%) in the control group and 6 in 4 of 21 patients (19%) in the implant group. A detailed categorization of all adverse events is provided in eResults 2 and eTable 2 in Supplement 2).

### Exploratory End Point Analyses

In the control group, 11 of 19 patients (58%) (95% CI, 34%-80%) received aVS rescue therapy (defined as intra-arterial vasodilator, percutaneous transluminal angioplasty, or induced hypertension) compared with 2 of 20 patients (10%) (95% CI, 1%-32%) in the implant group (*P* = .002). Computed tomography imaging between days 13 and 15 after aneurysm rupture revealed DCI in 6 of 19 patients (32%) (95% CI, 13%-57%) in the control group compared with 2 of 20 patients (10%) (95% CI, 1%-32%) in the implant group (*P* = .13). The mean length of intensive care unit stay was 30.2 days (95% CI, 22.9-37.4 days) in the control group and 20.8 days (95% CI, 13.9-27.6 days) in the implant group (*P* = .06) (eFigure 1 in Supplement 2). The mean duration of hospitalization was 45.0 days (95% CI, 27.3-62.7 days) in the control group and 29.3 days (95% CI, 12.5-46.0 days) in the implant group (*P* = .20) (eFigure 2 in Supplement 2). In patients who received the implant, the maximal median plasma concentration of nicardipine assessed until day 21 was 1.27 ng/mL (95% CI, 1.01-1.91 ng/mL) (eFigure 3 in Supplement 2). At 52 weeks, 12 of 18 patients (67%) in the control group and 16 of 19 patients (84%) in the implant group had reached a favorable outcome (*P* = .27) (eTable 3 in Supplement 2). Additional exploratory end points regarding functional, mortality, quality-of-life, and neuropsychological outcomes did not differ between groups (eResults 3 in Supplement 2). Monitoring of concomitant medications, including oral nimodipine, revealed no difference between groups.

## Discussion

The findings of this multicenter RCT demonstrate the safety and efficacy of applying a local vasodilator in addition to standard of care for the prevention of aVS after aSAH compared with standard-of-care treatment alone. The nicardipine release implant may represent a novel treatment strategy to prevent aVS and DCI, reduce therapeutic intensity, and shorten hospitalization times, which in turn may improve outcomes and provide an economic health care benefit for patients with aSAH.

The prophylactic use of calcium antagonists in patients with ruptured intracranial aneurysms for improving outcomes after aSAH remains only moderate, so improved treatment strategies are needed.^11^ However, phase 3 of the NEWTON (Nimodipine Microparticles to Enhance Recovery While Reducing Toxicity After Subarachnoid Hemorrhage) trial on the intrathecal application of nimodipine-releasing nanoparticles in aSAH was halted because the study was unlikely to meet its primary end point. Although the reasons seem to be multifactorial, a possible cause may have been changes in the cerebrospinal fluid dynamics after aSAH and the resulting lack of drug distribution within the basal cisterns.^12,13^ In addition, nimodipine is hydrophilic and thus systemically absorbed, which can dilute a localized treatment effect after intrathecal application. Nicardipine implants, on the other hand, represent a sustained, lipophilic drug distribution vehicle that is not systemically absorbed for a highly localized nicardipine delivery at the target site most likely to be at risk for aVS. Furthermore, the implants do not increase systemic nicardipine levels,^5,6^ so the risk for systemic adverse effects appears to be low, as supported by our group’s previous and present findings that plasma levels of nicardipine remained below a pharmacologically active level.^6^

Our positive findings regarding the effectiveness of nicardipine implants for the prevention of aVS fall in line with a previous study on the compassionate use of local nicardipine prolonged-release implants in aSAH,^9^ but unlike this previous nicardipine compound, the compound we used was produced according to a GMP-compliant manufacturing process and currently remains the only nicardipine implant in development.^14^

Although this RCT was not powered to permit a definite conclusion regarding outcome, the overall outcome compared favorably with previous RCTs on clipping of ruptured aneurysms,^15^ and both groups continued to exhibit clinical improvement until week 52. This conclusion is noteworthy considering that all patients in our cohort experienced moderate- to high-grade aSAH and were, by design, relatively young and without relevant comorbidities (eg, cardiovascular, oncologic). Another observation was that nicardipine implants reduced the need for aVS rescue therapies, which led to less clinical deterioration among patients in the implant group and decreased therapeutic intensity and procedure-related risk exposure.^16^ Although not statistically significant, our findings regarding the magnitude of the intensive care unit stay in the implant group fall in line with this observation and may justify the use of nicardipine implants from an economic health care standpoint.

### Limitations

As a main technical limitation, the nicardipine release implant is still limited to patients who require microsurgical treatment of ruptured aneurysms. Such patients also appear to be at a higher risk of developing aVS^14^; thus, nicardipine implants could provide them a safe and effective treatment solution. In the meantime, the increasing number of patients with aSAH who are treated endovascularly might put the practical relevance of using nicardipine implants into question.^15^ However, there are several countries where endovascular treatment is still prohibitively expensive, and there also will always be certain ruptured aneurysms that will require microsurgical treatment, for which nicardipine implants may play an important role. Moreover, a large RCT in aSAH has recently shown that approximately 49% of patients with ruptured aneurysms recruited by academic neurovascular centers in Germany, Switzerland, and Canada continue to be selected for microsurgical clipping, which underscores the relevance of microsurgery for the treatment of ruptured aneurysms.^17^ Another limitation is the low ethnic and racial diversity of our study population, which could be improved in a future phase 3 trial by participation of a higher number of centers with greater geographic spread.

## Conclusions

The findings of this RCT show that nicardipine implants placed during microsurgical aneurysm repair provide safe and effective prevention of moderate to severe aVS after aSAH. A phase 3 clinical trial to investigate the effect of nicardipine implants on clinical outcomes is warranted.

## Notes

Supplement 1.
Trial Protocol

Supplement 2. **eMethods 1.** Protocol Synopsis **eMethods 2.** Trial Visit Schedule **eResults 1.** Patient Disposition **eTable 1.** Patient Disposition at Visit 7 **eResults 2.** Adverse Events **eTable 2.** Adverse Events Overview (Safety Population) **eResults 3.** Exploratory End Point Analysis **eTable 3.** Functional Outcome at Week 52 **eFigure 1.** Length of ICU Stay **eFigure 2.** Duration of Hospitalization **eFigure 3.** Maximum Plasma Concentration (c_max_) of Nicardipine

Supplement 3.
Data Sharing Statement
