# Supplementary material for: Localized Nicardipine Release Implants for Prevention of Vasospasm After Aneurysmal Subarachnoid Hemorrhage: A Randomized Clinical Trial
Source: JAMA Neurol. 2024 Aug 19;81(10):1060–5. doi: 10.1001/jamaneurol.2024.2564 (PMC11334004; doi:10.1001/jamaneurol.2024.2564)
Supplement: Supplement 1. — Trial Protocol [file jamaneurol-e242564-s001.pdf]

STUDY PROTOCOL

TABLE OF CONTENTS

1 BACKGROUND INFORMATION AND STUDY RATIONALE 3

2 STUDY OBJECTIVES AND ENDPOINTS 7

3 STUDY DESIGN 11

4 SUBJECT SELECTION CRITERIA 13

5 CONCOMITANT MEDICATIONS 15

6 TREATMENT(S) 16

7 STUDY PLAN 19

8 STUDY PROCEDURES / EVALUATIONS 22

9 ADVERSE EVENT REPORTING AND HOSPITALISATION 30

10 DATA MANAGEMENT 34

11 STATISTICAL ANALYSIS PLAN 34

12 ETHICS COMMITTEE / IRB APPROVAL 38

13 REGULATORY REQUIREMENTS 39

14 INFORMED CONSENT 39

15 DIRECT ACCESS TO SOURCE DOCUMENTATION / DATA 41

16 STUDY MONITORING 41

17 QUALITY ASSURANCE 41

18 INSURANCE 42

19 CONFIDENTIALITY 42

20 PREMATURE TERMINATION OF THE STUDY 42

21 RECORD RETENTION 42

22 PUBLICATION OF RESULTS 43

23 DATA SAFETY MONITORING BOARD (DSMB) 43

24 STEERING COMMITTEE 44

25 REFERENCES 45

26 APPENDICES 47

BACKGROUND INFORMATION AND STUDY RATIONALE

Aneurysmal subarachnoid haemorrhage (aSAH) is associated with high morbidity and mortality with an incidence of 5-9 per 100,000 population. The incidence of aSAH peaks amongst those of 40-60 years[1, 8]; aSAH carries a high overall mortality rate of up to 67% [2, 8] with only around half of the survivors able to live independently. Given the age-related incidence and high morbidity and mortality, SAH has a high burden on society [3].

Conventionally following SAH, treatment is directed to securing the aneurysm to prevent further re-bleed. This however does not improve morbidity and mortality due to the haemorrhage. The only approved effective treatment to reduce morbidity is nimodipine [4]; its effects are small and despite its use, poor outcomes remain a significant problem [5]. Even in survivors conventionally considered to have made a good recovery, neurocognitive deficits are common, leading to extensive problems with social reintegration and functioning [6].

NicaPlant® is a modified release formulation of the calcium channel blocker nicardipine free base, and it is presented in the form of a rod shape implant (2 mm x 10 mm, 4 mg nicardipine load). The excipients used in the formulation for sustained release are a mixture of two completely degradable and fully synthetic (no animal sourced) polymers (Poly(D,L-lactide-co-glycolide), PLGA), with different molecular weights composed of a 50 : 50 ratio of lactate and glycolate.

NicaPlant® will be used to treat patients affected by aSAH at the time of aneurysm closure by surgical clipping.

In these patients, NicaPlant® will be placed in close proximity to the blood vessel walls within the basal cisterns at the time of the microsurgical aneurysm treatment, in order to locally deliver the active pharmaceutical ingredient (API) and prevent cerebral vasospasm (CV), cerebral infarction and delayed ischemic neurological deficit (DIND). CV and DIND are known to be major causes of death following aneurysm treatment and to be responsible for poor long term prognosis and disability in patients surviving aSAH [1, 8].

This procedure has already been successfully performed with a pharmaceutical equivalent known as nicardipine prolonged-release implants (NPRI) in over 250 aSAH patients. Up to 12 implants were used demonstrating excellent safety and tolerability profiles with no complications reported and no signs of neuronal toxicity or chemical meningitis [9, 10, 11, 12, 13]. All these studies demonstrated prevention of CV in vessels adjacent to the site(s) of implantation and decreased cerebral infarction and DIND. However, most of these studies lacked a control group.

NicaPlant® is a pharmaceutical equivalent of the NPRI; it has the same shape and size, same nicardipine free base load, similar excipient composition and comparable *in vitro* release profile.

In a recent phase IIa study the safety and tolerability of increasing doses (3, 6, 10 and 13 implants in 2, 2, 3 and 3 patients, respectively) was tested.

Eight serious adverse events occurred during the study that can be easily explained by the severe status of the patients and the major surgery. Five serious adverse events were classified as possible or probable related to the treatment either because of the lack of other explanations or, even if other explanations were more likely, a connection to the implants was still supposable. The variety of adverse events that can be suspected, already by developing a subarachnoid haemorrhage and the following clip ligation, and especially by reason of the small group a statistical evaluation of adverse reactions is not expedient.

The results of the phase IIa study overall demonstrate that NicaPlant® was safe and well tolerated. No obvious advantage from an exposure perspective could be observed with 13 versus 10 implants. More details of this study are presented in the Investigator's Brochure.

Investigational agent

NicaPlant® is a nicardipine modified release formulation, it is presented in the form of a rod shaped implant (2 mm x 10 mm, 4 mg nicardipine load).

Preclinical data

The prevention of cerebral vasoconstriction in dogs with subarachnoid haemorrhage by nicardipine-releasing implants comparable to NicaPlant® has been demonstrated in different published investigations. In addition, under conditions of global cerebral ischaemia, nicardipine was shown to improve cerebral blood flow and to prevent ischaemic cerebral tissue damage and improve recovery from damage in dogs, rats and gerbils *in vivo*. Furthermore, the pharmacodynamic activity of nicardipine as vasodilating agent has been demonstrated in various animal models as general vasodilatation and as vasodilatation with increase of blood flow in the arterial beds of different organs in rats, dogs and monkeys.

Secondary pharmacodynamic effects of nicardipine were seen as anti-oxidant and anti-neuroinflammatory activities, activities which may contribute to the therapeutic effect.

Published investigations on safety pharmacology demonstrated, in accordance with the investigations on primary pharmacology, a general improvement of blood flow in the major organ systems such as heart, central nervous system, gastrointestinal tract and kidneys. Under conditions of experimental infarction in the heart or central nervous system, nicardipine reduced the infarct size and improved tissue recovery as well as revascularisation. Besides a reduction of renal blood flow in hypertensive rabbits, which was not seen in normotensive rabbits, no adverse effects of nicardipine were seen in safety pharmacology studies.

Most probably based on the improved blood flow in major organs, nicardipine may reduce the toxicity of other drugs.

NicaPlant® brain local tolerance has been assessed in mice using implant slices. No development of local reactions was observed by intravital microscopy and by immunohistochemistry, while occurred the expected vasodilation.

Full details of the preclinical data are described in the Investigator's Brochure.

Risks / benefits

The potential therapeutic benefits of NicaPlant® outweigh any potential risks for patients with aSAH. NPRIs have already demonstrated good safety and tolerability profiles with no drug-related adverse events; in particular, there have been no observed significant differences in the incidence of central nervous system infection or hydrocephalus. Furthermore, all previous NPRI studies demonstrated prevention of CV in vessels adjacent to the site(s) of implantation and decreased cerebral infarction and DIND.

NicaPlant® is a pharmaceutical equivalent of the NPRI. It has the same shape and size, meaning the implantation procedure remains unchanged in risk profile from previous studies. The API (nicardipine free base) load is unchanged with a similar excipient composition leading to a comparable *in vitro* release profile.

Overall NicaPlant® was safe and well tolerated in the Phase IIa study. The patient number per group was too small to perform differential statistics but regarding the current safety data available, the potential therapeutic benefit still outweighs the potential risks. The implants have proven to be easy to implant, as stated by investigators, and the DSMB assessed the benefits and risks at every meeting before allowing the progression to the next dosing level. In the DSMB's final meeting, after reviewing adverse events and pharmacokinetics, the board decided that there seems to be no additional benefit after the dose progression from 10 to 13 implants.

The proposed clinical study is a randomised, single-blind, parallel-group design comparing NicaPlant® implants versus standard of care in 40 patients who have had aSAH.
The two groups of patients will be subjected to the following treatments:

- Standard of care plus NicaPlant®.
- Standard of care.

Both groups of patients will receive the standard of care according to treatment guidelines [8] and all further medical care will not be different between the groups. Patients in the interventional group will only in addition receive NicaPlant® implants.

NicaPlant® will be administered by placement during microsurgical clipping of the ruptured aneurysm in proximity to all of the exposed cerebral blood vessels.

Patients will be assessed to determine the efficacy of NicaPlant® application to reduce the occurrence of cerebral vasospasm and to prevent early morbidity/mortality following aSAH. Cerebral vasospasm will be assessed by analysis of angiograms to determine narrowing of the proximal blood vessels. A cerebral vasospasm is defined as lumen reduction ≥33%

Safety assessments will be made to confirm the safety and tolerability of the selected dose of NicaPlant® up to patient discharge, or day 21, whichever occurs first.

Clinical Outcomes will be recorded at 12 weeks and 1 year (week 52) after the aSAH.

A data safety monitoring board (DSMB) will be set-up to monitor safety throughout the trial period and provide recommendations for any necessary actions. A steering committee will receive and review the reports from the DSMB and take action as appropriate.

The final analysis will be carried out when the final patient completes the week 12 assessment. Data collected for patients at Week 52 after aSAH will be analysed and included as an addendum to the final report.

Given that NicaPlant® is implanted at the time of aneurysmal clipping, it should be emphasised that the investigational product cannot be withdrawn after implantation.

In summary, published data on the safety and effectiveness of NPRIs supports the premise that NicaPlant® offers significant potential therapeutic benefits, without significant clinical risk.

Furthermore, the results of the phase IIa study involving a total of 10 actively treated patients (with 2 patients each in 3 and 6 Implant groups and 3 patients each in 10 and 13 Implant groups) demonstrate that NicaPlant® was safe and well-tolerated up to 13 implants/ patient.

Study Rationale

This is a Safety, Tolerability, Efficacy and Pharmacokinetic study of NicaPlant® in Subarachnoid Haemorrhage involving two treatments groups, standard of care and those receiving standard of care and the investigational product NicaPlant®. Those randomised to the investigational group will receive NicaPlant® in order to improve and reduce the long-term complications of aSAH such as CV & DIND.

The objective is to assess the efficacy of NicaPlant® in preventing the occurrence of angiographic cerebral vasospasm following aSAH and its complication till patient discharge or day 21, whichever occurs first; clinical outcomes will be assessed by rating scales at 12 and 52 weeks.

Dose rationale

Based on the results of the phase IIa clinical study performed using NicaPlant® and previous studies performed with the NPRIs, it is expected that the NicaPlant® efficacious dose will be of 10 implants (total 40 mg modified release nicardipine free base).

In the phase IIa, following aneurysm clip ligation, the predetermined number of NicaPlant® implants (i.e. doses of 3, 6, 10 or 13 implants in a total of 10 patients: 2, 2, 3 and 3 patients, respectively) were implanted into the basal cisterns, in direct contact with the exposed cerebral blood vessel walls.

The distribution of the implanted polymers followed a pre-defined protocol, dependent on aneurysm location to achieve maximum delivery of nicardipine to the vessel segments at highest risk of vasospasm and was defined for the different number of implants in the different cohorts in the study. An independent DSMB reviewed the unblinded safety data between cohorts and expressed consent to progress to the next cohort.

In the phase IIa study oral nimodipine (normally administered as standard of care for aSAH) was replaced by placebo in the interventional groups as its side effects could have masked possible NicaPlant® systemic side effects. The phase IIa study demonstrated minimal systemic exposure, with nicardipine plasma levels below pharmacological active levels (Cmax for the 10 implants cohort was: 1.9± 0.5 ng/mL over the period from 0 – 21 days, with

values for the three patients in this group being 1.39, 1.91 and 2.41 ng/mL), and with no signs of any of its known systemic side effects (no hypotension, no elevation of liver enzymes). The safety and tolerability of up to 13 implants of NicaPlant® was demonstrated, however no clear advantage in term of exposure could be observed with 13 versus 10 implants. Therefore, in the current phase IIb study 10 NicaPlant® implants will be administered in addition to standard of care.

Trial conduct

This study will be conducted in compliance with the protocol and according to Good Clinical Practice and applicable regulatory standards. No deviation from the protocol will be implemented without the prior review and approval by the relevant ethics and regulatory authorities, except where it may be necessary to eliminate an immediate hazard to a research subject. In such case, the deviation will be reported to the relevant ethics and regulatory authorities as soon as possible.

Population

The population to be studied are patients with spontaneous aSAH, World Federation of Neurological Surgeons (WFNS) grade III-IV, who present within 48 hours of ictus and that clip ligation of the aneurysm is achievable within 72 hours of aSAH (in the opinion of the Investigator).

Patients with WFNS grade I, II and V are excluded from this study.

This decision is based on the recent publication of results of the NEWTON 2 study, that investigated the activity of EG-1962, microparticle containing nimodipine and delivered via an EVD to aSAH patients, indicated that the compound even if able to significantly reduce occurrence of CV compared to the control group, had no effect in improving the percentage of patient presenting a 90 days good clinical outcome in grade I and II patients (GOSE 6-8; EG-1962: 46%, control: 55%), therefore posing only potential additional risks for these patients (D Hänggi et al., for the NEWTON 2 Investigators, International Stroke conference, 6-8, Feb, 2019, Honolulu, Hawaii).

STUDY OBJECTIVES AND ENDPOINTS

Primary objective

- To assess the efficacy of local nicardipine application via controlled-release polymers (NicaPlant®) in preventing the occurrence of cerebral vasospasm following aneurysmal Subarachnoid Haemorrhage (aSAH), by evaluating the incidence of moderate to severe cerebral angiographic vasospasm assessed by digital subtraction angiography (DSA).
- To assess the safety and tolerability of the selected dose of NicaPlant® (10 implants).

Secondary objectives

- To assess the efficacy of local nicardipine application via controlled-release polymers (NicaPlant®) on:

Incidence of new cerebral infarcts on CT of the brain performed at Day 14 ± 1 post-aneurysm rupture, or before patient discharge, versus post-intervention (i.e. post clip ligation and NicaPlant® implantation) CT scan.

Exploratory objectives

- To assess the efficacy of local nicardipine application via controlled-release polymers (NicaPlant®) on:

Incidence of vasospasm-related morbidity/mortality within 21 days post-aneurysm rupture or before patient discharge, defined by at least one of the following:

  - DIND.
  - Death caused by vasospasm, delayed ischemic neurological deficit (DIND), infarcts or complications due to anti-vasospasm therapy.
  - The need for anti-vasospasm rescue therapy before patient discharge or within 21 days post-aneurysm rupture.

- To assess the pharmacokinetic profile of NicaPlant® (local nicardipine application via polymers) in plasma and its levels in CSF (only in patients provided with an EVD at time of CSF removal for medical reasons.
- To assess the effect on clinical outcome and mortality following aSAH at week 12.
- To assess the effect on clinical outcome and mortality following aSAH at Week 52.

Primary Outcome Endpoints

The primary outcome endpoints are:

Safety:

Daily assessments:

- Adverse events (AEs)
- Shunt-dependent hydrocephalus (to Day 21±1 or patient discharge)
- Bacterial meningitis (to Day 21±1 or patient discharge)
- Change in grading of AE severity

At the defined visits 1 to 5:

- Vital Signs: blood pressure, pulse rate, respiratory rate and body temperature
- Electrocardiogram (ECG)
- Full blood count, urea and electrolytes, liver function tests, C-reactive protein

Safety assessments will be made from baseline until discharge or day 21±1 and at all subsequent visits. Adverse Events occurring after day 21±1 will be collected but will no longer be considered treatment emergent.

Efficacy:

Primary efficacy endpoint

- Incidence of moderate to severe cerebral angiographic vasospasm after aneurysmal subarachnoid haemorrhage, where angiographic vasospasm is defined as a ≥33% reduction in diameter in at least one vessel segment by comparison to preoperative angiography.

Secondary Outcome Endpoints

The secondary outcome endpoints are:

- Incidence of new cerebral infarcts on CT scan at day 14 ± 1, or before patient discharge (compared with postoperative CT scan).

Exploratory Outcome Endpoints

The exploratory outcome endpoints are:

- Incidence of vasospasm-related morbidity/mortality within 21 days or before patient discharge, defined by at least one of the following:
  a. DIND.
  b. Death caused by vasospasm, delayed ischemic neurological deficit (DIND), infarcts or complications due to anti-vasospasm therapy.
  c. The need for anti-vasospasm rescue therapy.
- Length of Intensive Care Unit (ICU) stay
- Length of Hospital Stay
- Clinical outcome at Week 12 post-aneurysm rupture as measured by:
  a. Modified Rankin Scale (mRS) – “Good Outcome” defined as mRS of 0 to 2
  b. Glasgow Outcome Scale (Extended) (GOSE) – “favourable outcome” of 6-8, “unfavourable outcome” of 0-5
  c. Montreal Cognitive Assessment tool (MoCA) - score 0-30 higher better
  d. Health related quality of life assessed using the EQ-5D-5L and SF36.

- Long-term Clinical outcome at Week 52 post-aneurysm rupture as measured by:
    a. Modified Rankin Scale (mRS) – "Good Outcome" defined as mRS of 0 to 2
    b. Glasgow Outcome Scale (Extended) (GOSE) – "favourable outcome" of 6-8, "unfavourable outcome" of 0-5
    c. Montreal Cognitive Assessment tool (MoCA) - score 0-30 higher better
    d. Health related quality of life assessed using the EQ-5D-5L and SF36.

Pharmacokinetics (PK):

Blood sampling for PK assessment will be carried out in all patients on day of clip ligation (0, 6, 12 and 24 hours post clip ligation, ±20 minutes each), day 8, day 14 after aneurysm rupture and before discharge or day 21 (whichever occurs first). Blood sampling for PK assessment will additionally be done every time blood is drawn for medical reasons up to patient discharge or day 21 but only once per day.
Single cerebrospinal fluid (CSF) samples obtained from patients provided for medical reasons with an external ventricular drain (EVD) will be collected every time a CSF sample is removed for medical reasons and up to patient discharge or to day 21, to determine nicardipine levels.
Only samples collected in patients treated with NicaPlant® will be analysed at the end of this study.
Where possible, the following PK parameters will be determined:

- Maximum plasma concentration (C_{max}), obtained directly from the observed concentration versus time data

- Time of maximum plasma concentration (t_{max}), obtained directly from the observed concentration versus time data

- Area under the plasma concentration time curve from zero (pre-dose) extrapolated to infinity ($AUC_{0\text{-}\infty}$), calculated by linear up/log down trapezoidal summation and extrapolated to infinity by addition of the last quantifiable concentration divided by the elimination rate constant: $AUC_{0\text{-}t} + C_{last}/\lambda_z$. If the extrapolated area (C_{last}/λ_z) is greater than 20% of $AUC_{0\text{-}\infty}$, then $AUC_{0\text{-}\infty}$ and related parameters may not be reported

- Area under the plasma concentration-time curve during the dosing interval ($AUC_{0\text{-}t}$) in Part B only, calculated by linear up/log down trapezoidal summation

- Half-life ($t_{1/2}$, h). Visual assessment will be used to identify the terminal linear phase of the concentration-time profile. A minimum of 3 data points will be used for determination

- Apparent systemic clearance (CL/F, L/h)
- Apparent volume of distribution (V_z/F, L)
- The time interval (h) of the log-linear regression to determine $t_{1/2}$ ($t_{1/2}$, Interval)
- Number of data points ($t_{1/2}$, N) included in the log-linear regression analysis to determine $t_{1/2}$
- Coefficient of determination (Rsq) for calculation of λ_z. If Rsq is less than or equal to 0.800, λ_z and related parameters will not be reported
- Percentage of AUC obtained by extrapolation (%AUC_{ex})

Pharmacodynamics (PD):

The pharmacodynamic endpoints are incidence of moderate to severe angiographic measurements of cerebral vasospasm and new cerebral infarcts as assessed by the independent neuroradiologist, and DIND.

STUDY DESIGN

Overall study design and plan description

This study is a randomised, single-blind, parallel-group design comparing 10 NicaPlant® implants / patient versus standard of care in overall 40 patients who have had aSAH.

The two groups of patients will be subjected to the following treatments:

- Standard of care plus NicaPlant®.
- Standard of care.

Both groups of patients will receive the standard of care according to treatment guidelines [8] and all further medical care will not be different between the groups. Patients in the interventional group will only in addition receive NicaPlant® implants.

NicaPlant® will be administered as per the implantation protocol, (in brief: by placement during microsurgical clipping of the ruptured aneurysm in proximity to all of the exposed cerebral blood vessels).

Patients will be assessed to determine the efficacy of NicaPlant® application to reduce the occurrence of angiographic cerebral vasospasm and to prevent early morbidity/mortality following aSAH. Cerebral vasospasm will be assessed by analysis of angiograms to determine narrowing of the proximal blood vessels.

Clinical Outcomes will be recorded at 12 weeks and 1 year (52 weeks) after the aSAH. Safety assessments will be made to confirm the safety and tolerability of the selected dose of NicaPlant®.

A data safety monitoring board (DSMB) will be set-up to monitor safety throughout the trial period and provide recommendations for any necessary actions. A steering committee will receive and review the blinded reports from the DSMB and take action as appropriate.

The DSMB will convene after 20 patients have been recruited and monitored to discharge, or day 21, whichever occurs earlier.

The final analysis will be carried out when the last patient completes the week 12 assessment, the database has been locked at this time point and unblinding has occurred. Data collected for patients at Week 52 after aSAH will be analysed and included as an addendum to the final report.

Discussion of study design, including the choice of control groups

Randomisation

This is a randomised, single blind study. Patients will be randomised to either the standard of care plus treatment group or standard of care group. Therefore, twenty patients will receive standard of care plus 10 NicaPlant® implants, and twenty will be assigned to the standard of care control group.

Blinding

The study design does not include a placebo implant for ethical reasons; therefore, it is not possible to blind the neurosurgeons performing the aneurysmal clipping procedure to study treatment. Accordingly, the standard of care control group will receive no intracranial treatment (i.e., no placebo implants).

Patient selection bias is controlled by randomisation; patients will be blinded to which treatment they are receiving (implant or standard of care group) throughout the study i.e. the study is single blinded.

Therefore, the treatment groups are as follows:

- Patients receiving standard of care and NicaPlant® implants.

OR

- Patients in standard of care.

Patients will be assigned a corresponding randomisation code at consent; in the operation room, following aneurysm clip ligation, a member of the unblinded operation room team will verify the randomisation code using the IWRS system; if the patient is to receive NicaPlant® implants the implantation procedure goes ahead; if not, no implantation is carried out.

In emergency situations, when the identity of the treatment arm must be made known, the treatment arm of the patient will be available to designated and authorised personnel using the IWRS system. Access to the IWRS is held by the responsible neurosurgeon and other

designated authorised site personnel, to be agreed prior to study commencement. Full details of the randomisation including user access will be provided in the study IWRS user guide.

Study team staff (including those undertaking Safety Assessments, TCD readings, mGCS assessments and Clinical Outcome assessments) will be blinded to the treatment. An independent specialist, also blinded to the treatment, will undertake the analysis of the angiograms and computed tomography imaging of the brain for the diagnosis of cerebral vasospasm and new cerebral infarcts, respectively, for study endpoint assessment.

Trained personnel collecting data on clinical outcome at week 12 and 52 will also be blinded. In cases of DIND or anti-vasospasm rescue therapy as determined by the sites, all required data (e.g. clinical data, case narratives, angiograms, CTs) will be provided to a board of blinded independent experts to adjudicate on the presence of DIND or anti-vasospasm rescue therapy.

SUBJECT SELECTION CRITERIA

Subject recruitment

Patients admitted with a diagnosis of spontaneous aSAH will be assessed by the study team against the inclusion and exclusion criteria. Identified subjects who fulfil the criteria will be approached by a designated medical member of the study team, who will in turn obtain consent according to the informed consent procedure (see section 0 INFORMED CONSENT).

Inclusion criteria

1. Austria: Informed consent is obtained from patients with capacity. If patients lack capacity, informed consent is obtained from a personal legal representative. If no suitable personal legal representative is available and the treatment needs to be given urgently, written informed consent will be obtained as soon as the participant is responsive or a representative is available.

   Germany: An independent physician is to be consulted to confirm that the presumed will of the patient on the participation in the study has been determined and respected. Written informed consent will be obtained as soon as the participant regains capacity.
2. Male or female patients aged 18 to 75 years (inclusive).
3. World Federation of Neurological Surgeons (WFNS) grade III-IV.
4. Ruptured saccular aneurysm, confirmed by angiography.
5. Onset of aSAH clinical symptoms within the preceding 48 hours.
6. Treatment of aneurysm via surgical clip ligation within 72 hours of aSAH is achievable.
7. Female patients of child-bearing potential must have a negative pregnancy test (urine or serum) at screening and must agree to use adequate birth control up to 12 weeks after implantation of the study drug. Female patients are considered to be not of child-bearing potential if they have a history of tubal ligation or hysterectomy or are post-menopausal with a minimum of 2 years without a natural menstrual cycle. Male

patients must agree to use adequate birth control up to 12 weeks after implantation of the study drug.

Exclusion criteria

1. SAH due to other causes (e.g. trauma, fusiform or mycotic aneurysm).
2. World Federation of Neurosurgery (WFNS) grade I, II and V patients.
3. Pregnant or Lactating Women.
4. Intraventricular or intracerebral blood, in the absence of subarachnoid blood.
5. Treatment of aneurysm via endovascular embolisation.
6. Presence of moderate or severe vasospasm on screening angiography.
7. Any known or CT /MRI evidence of previous major cerebral damage
8. Evidence of a cerebral infarction with neurological deficit on pre-treatment CT/MRI.
9. History of malignant disease (except for non-melanoma skin cancer) within the previous 5 years or any history of malignant brain tumours or brain metastasis.
10. Patients who have received an investigational product or participated in another interventional clinical study within 30 days prior to randomisation.
11. Patients with known allergy for Poly(D,L-lactide-co-glycolide) (PLGA) or nicardipine.
12. Major complication during aneurysm repair such as, but not limited to, massive intraoperative haemorrhage, brain swelling, or inability to secure the ruptured aneurysm.

Subject withdrawals

In all circumstances, patients and/or personal legal representatives should be made aware of the rights to refuse participation in a clinical trial and are entitled to freely withdraw their informed consent, without giving reasons. Patients and/or personal legal representatives should be assured that the withdrawal from the trial will not cause prejudice, will not result in any determinant and will not affect treatment. In addition, refusal to give consent or withdrawal of consent to participate in research must not lead to any liability or discrimination (e.g., with regard to insurance or employment) against the person concerned.

The Investigator also has the right to withdraw subjects from the study in the event of:

- Use of any concomitant medication specified as not permitted
- Noncompliance with the protocol and/or lack of willingness or commitment to co-operate in all phases of the study
- Protocol deviations
- Pregnancy after entry into study
- Adverse event which is considered intolerable by the patient and/or legal representative

- Intercurrent illness that necessitates pharmacological treatment with a drug which interacts in any way with the test treatment
- Development of an exclusion criterion

Should a patient and/or personal legal representative decide to withdraw for other reasons, all efforts will be made to complete and report the observations as thoroughly as possible. A complete final evaluation at the time of withdrawal will be performed with an explanation of the exact reason why the subject is withdrawing from the study.

The Investigator is responsible for the optimal individual treatment of the patient.

The Investigator must fill in the “Study termination” in the case reporting form (CRF) explaining all reasons for withdrawal.

After a patient withdraws from the trial, the Investigator is still responsible for reporting SAEs considered causally related to the study drug as well as any pregnancy and outcome of the pregnancy. In addition, the Investigator needs to ensure appropriate treatment and follow-up of each adverse event still ongoing at the time of the patient’s discontinuation.

No reproductive toxicology or embryo/foetal development studies have been performed with NicaPlant®. The product will not be used in pregnant women.

Replacement of Withdrawn Patients

Patients that withdraw prior to hospital discharge, or day 21, whichever occurs first, will be replaced. Replacement patients will receive the same treatment as the withdrawn patient. A maximum of 10 replacements will be done.

Patients who die will be considered to have completed the study and will not be replaced.

CONCOMITANT MEDICATIONS

Permitted concomitant medications

Concomitant treatment will be permitted unless its use is contraindicated or there are significant interactions with NicaPlant®.

Non-permitted concomitant medications unless indicated

NicaPlant® is contraindicated in those who are hypersensitive to any component of a nicardipine-containing product.

A list of non-permitted concomitant medications will be provided to sites based on available information on the known interaction with nicardipine.

**Drug class:**	**Specific active substance (if applicable)**	**Effect on Nicardipine:**

CYP450 3A4 Inducers	Carbamazepine<br>Rifampicin	Alter the plasma levels of nicardipine.
CYP450 3A4 Inhibitors	Cimetidine<br>Grapefruit Juice	Alter the plasma levels of nicardipine.
	Cyclosporine<br>Tacrolimus<br>Sirolimus	Elevated levels of cyclosporine, tacrolimus or sirolimus in the plasma.
Calcium Ion Antagonist	Cardene®	Careful monitoring of serum digoxin levels as levels could be increased.
Beta-Blocker	Fentanyl Anaesthesia with concomitant use of Beta-Blocker and Calcium Blockade	Severe Hypotension
Anti-Hypertensives		Postural Hypotension

TREATMENT(S)

Appearance and content

Investigational Medicinal Product

NicaPlant® is a nicardipine modified release formulation, it is presented in the form of a rod-shaped implant (2 mm x 10 mm, 4 mg nicardipine load).

NicaPlant® will be given on top of the standard of care-treatment as determined at the sites according to the treatment guidelines. [8]

A main difference to the previous study is the lack of limitations for an additional treatment with Nimodipine or any other treatment needed according the treatment guidelines since the safety of patient should not be reduced. Furthermore, the sovereignty of the study sites will not be subducted by the study since patients in emergency situations within the intensive care unit need an individual therapy adapted to their needs and with no delays.

Dosage and administration

Dosage

NicaPlant® will be administered as per the implantation protocol. The selected dose is 10 implants/ patient.

Administration

Following aneurysm clip ligation, the NicaPlant® implants will be placed into the basal cisterns, in direct contact with the exposed cerebral blood vessel walls.

The distribution of the implanted polymers will follow a pre-defined protocol dependent on aneurysm location to achieve maximum delivery of nicardipine to the vessel segments at highest risk of vasospasm.

Example Implantation Sites:

Anterior communicating artery aneurysm (A)

Middle cerebral artery aneurysms (B)

Internal carotid artery aneurysms(C):

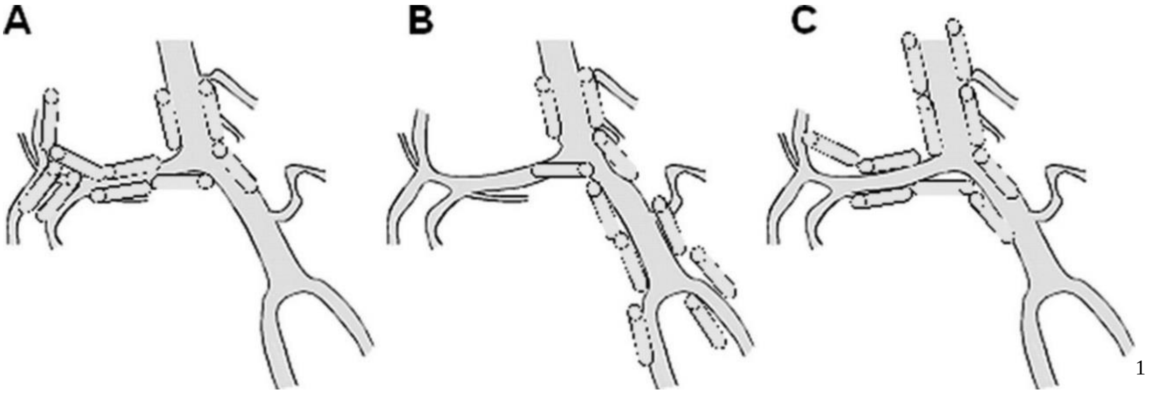

[1]

Sites of NicaPlant® placement are defined in the Implantation Protocol for all sites of saccular aneurysms.

Packaging

IMP is assembled for Qualified Person Certification and release for use in the clinical trial at the manufacturing company AMW GmbH, Birkerfeld 11, 83627 Warngau, Germany and delivered as 10 implants in lid covered boxes, placed into a pouch terminally gamma sterilised.

Labelling

All study medications will be labelled in accordance with Annex 13: Manufacture of investigational products and local requirements.

As a centralised randomisation system is used, the randomisation number of the patient will not be pre-printed on the label. After a patient is randomised to the Standard of care plus NicaPlant® group and received a randomisation code through the IWRS system, the code will be written onto the label of the used implant box by the site’s study team.

[1] Drawings from Barth M, Capelle HH, Weidauer S, et al., Effect of nicardipine prolonged-release implants on cerebral vasospasm and clinical outcome after severe aneurysmal subarachnoid haemorrhage: a prospective, randomised, double-blind phase IIa study, Stroke, 2007;38:330–6.

If there is an issue during implantation, like one or more dropped implants, a second implant box will be used for the patient and the randomisation code will be also written onto the label of the second implant box.

Blinding and randomisation

Randomisation

Up to 40 patients (20 will receive standard of care, 20 will receive standard of care and 10 NicaPlant® implants) will be included in the study.

Patients that withdraw prior to hospital dismissal will be replaced. Replacement patients will receive the same treatment as the withdrawn patient. A maximum of 10 replacements will be done. Patients who die will be considered to have completed the study and will not be replaced.

Patients are to be randomised at Visit 1 within 48 hrs of aneurysm rupture. A member of the study team will randomize patients in the IWRS system and will receive a randomisation code assigned to the patient. It is essential for all patients that a participation within the study is not causing any delay in the treatment and the safety and wellbeing of the patients needs to be the main focus all the time.

Blinding

Blinding is described in section 0.

Blinding will be recorded in a blinding log – on this log, blinded and unblinded study team members will be tracked. If there is an isolated break in the blind that/those member(s) of the research team must be transferred to the unblinded team.

For purpose of clarity, the un-blinded and blinded personnel is listed in the following table.

Un-blinded	Blinded
• Implantation operation room Team	• ICU team, undertaking Safety Assessments, TCD readings and GCS assessments and angiograms and computer tomography scans for patient management decisions.<br><br>• Independent specialist undertaking analysis of the angiograms and CT/MRI brain scans for the diagnosis of vasospasm and new cerebral infarcts.<br><br>• Trained personnel collecting data on clinical outcome and mortality week 12

	and week 52.<br>• Independent experts adjudicating on the presence of DIND and anti-vasospasm rescue therapy

Treatment compliance

NicaPlant® is implanted once (at the time of aneurysmal clipping); therefore, there are no treatment compliance issues.

Blood samples are collected for safety and pharmacokinetic measurements at fixed time points. Compliance will be monitored throughout the study.

Drug storage

All study medication will be stored at 2 - 8°C protected from light in the storage area, which will be a secure, temperature controlled, locked environment with restricted access.

No special procedures for the safe handling of NicaPlant® are required. The Sponsor will be permitted upon request to audit the supplies, storage, dispensing procedures and records provided that the blind of the study is not compromised.

Drug accountability

In accordance with GCP, the clinical unit will account for all supplies of NicaPlant®. Details of receipt, storage, assembly and return will be recorded.

All unused supplies of NicaPlant® will either be destroyed or returned to the study Sponsor at the end of the study in accordance with instruction by the Sponsor.

STUDY PLAN

Study Timings

Timing in the study is measured from time of aneurysm rupture; as the time of rupture cannot be determined precisely it shall be defined as the time from onset of first symptoms.

Continuous Assessment

The following assessments will be carried out on a continuous basis and as events arise:

Adverse Events and separately, bacterial meningitis and shunt-dependent hydrocephalus.

Visit 1: Pre-implantation assessment (within 48 hours of aneurysm rupture and SAH)

The following assessments will be performed and documented in all patients admitted with a diagnosis of spontaneous aneurysmal SAH and consented appropriately to the study:

- Inclusion/Exclusion Criteria (screening failures to be recorded)
- Angiography (DSA) of the ICA bilateral and both vertebral arteries
- CT of the brain
- Demographics/Medical History
- Physical examination
- Pregnancy test
- Vital signs (blood pressure, pulse rate, respiratory rate, temperature)
- ECG
- Haematology, serum biochemistry
- mGCS
- Randomisation
- Informed consent procedure according to section 14

The assessments for the participation in the study shall not cause any delay in the treatment of the patients. The safety and wellbeing of the patients needs to be the main focus all the time.

Visit 2: Clip Ligation & Implantation (within 72 hours of aneurysm rupture)

Eligible patients will be randomised to either NicaPlant® implantation or no implantation.

- Clip ligation
- Implantation (if randomised to the study group) of NicaPlant® implants
- PK Plasma sampling (0, 6, 12, 24 hrs after implant, ±20 minutes each)
- CSF sampling (patients with EVD and CSF sampling performed for standard of care only)
- CT brain scan, post-surgery, 36 ± 12 hours from clip ligation (on NicaPlant® application) to identify surgery related ischemic lesions and for baseline assessment.

Ongoing Assessments (day of surgery to day of patient discharge or day 21±1 post aneurysm, whichever occurs first)

- Daily – mGCS, TCD reading for evidence of vasospasm (to be confirmed by DSA), CSF sampling (patients with EVD in days when CSF sampling is performed for medical reasons only), PK Plasma sampling on days blood is drawn for medical reasons but only once per day (time of sampling to be recorded).

Visit 3: Angiography (DSA) (day 8±1 post aneurysm rupture or whenever vasospasm is suspected by sonographic or clinical means).

- Angiographic assessment for vasospasm (even if no clinical evidence is present); if the patient develops clinical or sonographic changes suggestive of vasospasm prior to day 8, an angiogram will be performed to confirm the vasospasm and the angiographic measurement will replace the one scheduled at day 8±1 (Visit3). Both ICA and the dominant vertebral artery should be documented.

- PK Plasma sampling (time of sampling to be recorded).
- vital signs (BP, pulse rate, respiratory rate, temperature), ECG, haematology, serum biochemistry

Visit 4: before patient discharge or day 14±1 post aneurysm rupture, whichever occurs first

- CT brain scan, for assessment of new cerebral infarcts.
- PK Plasma sampling (time of sampling to be recorded).)
- vital signs (BP, pulse rate, respiratory rate, temperature), ECG, haematology, serum biochemistry

Visit 5: day of patient discharge or day 21±1 post aneurysm, whichever occurs first

- PK Plasma sampling (time of sampling to be recorded).).
- vital signs (BP, pulse rate, respiratory rate, temperature), ECG, haematology, serum biochemistry
- Modified Rankin Scale (mRS)

Visit 6: Week 12 post aneurysm rupture

- Modified Rankin Scale (mRS)
- extended Glasgow Outcome Scale (GOSE)
- Montreal Cognitive Assessment tool (MoCA)

- Health related quality of life assessed using the EQ-5D-5L and SF36
- Mortality due to SAH

Visit 7: Week 52 post aneurysm rupture (Data to be reported in a CSR addendum)

- Modified Rankin Scale (mRS)
- extended Glasgow Outcome Scale (GOSE)
- Montreal Cognitive Assessment tool (MoCA)
- Health related quality of life assessed using the EQ-5D-5L and SF36
- Mortality due to SAH

STUDY PROCEDURES / EVALUATIONS

Safety Measurements

The following safety parameters will be recorded according to the trial protocol:

- Adverse events (AEs)
- Shunt-dependent hydrocephalus
- Bacterial meningitis
- Change in grading of AE severity
- Vital Signs: blood pressure, pulse rate, respiratory rate and body temperature
- Electrocardiogram (ECG)
- Full blood count, urea and electrolytes, liver function tests, C-reactive protein

Efficacy Measurements

The following efficacy parameters will be recorded according to the trial protocol:

- Incidence of moderate to severe cerebral angiographic vasospasm assessed by digital subtraction angiography (DSA) within at 8±1 days after aneurysm rupture where angiographic vasospasm is defined as a ≥33% reduction in diameter in at least one vessel segment by comparison to preoperative (pre NicaPlant® implantation) angiography and/or qualitatively assessed as moderate to severe vasospasm (in case the baseline measurement is missing). A day 8±1 angiogram will be performed even if the patient has no clinical or sonographic evidence of vasospasm.

  If the patient develops clinical or sonographic changes suggestive of vasospasm prior to day 8, and angiogram (DSA) will be performed to confirm the vasospasm and the angiographic measurement will replace the one scheduled at day 8±1. In case the patient develops clinical or sonographic changes suggestive of vasospasm after the

day 8±1 and prior to patient discharge or day 21, an angiogram (DSA) will be performed to confirm the vasospasm, unless this is not clinically indicated.

Secondary endpoint

1. Incidence of new cerebral infarcts on CT scan at day 14 ± 1, or before patient discharge (compared with postoperative CT scan).

Other exploratory endpoints

- Incidence of vasospasm-related morbidity/mortality within 14 days post-aneurysm rupture, or before patient discharge, defined by at least one of the following:
    a) DIND
    b) Death caused by vasospasm, delayed ischemic neurological deficit (DIND), infarcts or complications due to anti-vasospasm therapy
    c) The need for anti-vasospasm rescue therapy within patient discharge
- Daily assessment of modified Glasgow Coma Scale (mGCS) up to patient discharge or day 21.
- Clinical outcome at Weeks 12 & 52 post-aneurysm rupture as measured by:
    - Modified Rankin Scale (mRS)
    - Glasgow Outcome Scale (Extended) (GOSE)
    - Montreal Cognitive Assessment tool (MoCA)
    - Health related quality of life assessed using the EQ-5D-5L and SF36
    - Mortality due SAH

Pharmacokinetic (PK) and Pharmacodynamic (PD) Measurements

The following PK and PD parameters will be recorded according to the trial protocol:

PK:

PK blood sampling will be carried out in all patients on day of clip ligation at 0, 6, 12 & 24h post clipping and on days 8, 14 and day of discharge or day 21 after aneurysm rupture. Additionally PK blood sampling will be done when blood is collected for medical reasons but only once per day; only samples collected in patients treated with NicaPlant® will be analysed at the end of this study.

Whole blood samples (2 mL) will be collected into a Li-heparin tube and processed for plasma.

Samples will be collected, labelled, processed, stored and shipped as detailed in the Study Operation Manual.

Cerebrospinal fluid samples (500µL) will be collected in patients with EVD whenever CSF removal is performed for standard of care.

PD:

The pharmacodynamic endpoints are incidence of moderate to severe angiographic measurements of cerebral vasospasm and new cerebral infarcts as assessed by the independent neuroradiologist and DIND.

Definitions of Assessments

World Federation of Neurological Surgeons Grading System for Subarachnoid Haemorrhage (WFNS)

The WFNS is a 5-point scale that is a simple, reliable and clinically valid way to grade a patient with aSAH. This system offers less inter-observer variability than some of the earlier classification systems.

The grading is based on the Glasgow coma score (GSC see later description).

Grade 1	GCS score of 15 without focal deficit
Grade 2	GCS score of 13 or 14 without focal deficit
Grade 3	GCS score of 13 or 14 with focal deficit
Grade 4	GCS score of 7-12
Grade 5	GCS score of 3-6

Assessment of Cerebral Vasospasm and New Cerebral Infarct by a Centralised Neuroradiologist Efficacy Assessment.

All angiograms and computed tomography of the brain are to be evaluated locally for patient management decisions based on the local standard procedures.

For the assessment of the efficacy of NicaPlant® for the endpoint analysis, angiograms and computed tomography scans of the brain are to be analysed by an independent blinded neuroradiologist.

For this purpose, diameters of the proximal vessel segments (C1, M1, A1, P1, BA see diagram below) are to be measured in the baseline and the follow-up angiograms. To overcome potential bias from angiograms with different magnifications, the following procedure will be used:

1. The angiograms are to be optically magnified 5× to measure different vascular sections more precisely.
2. Measurements are performed an arterial filling image in the lateral view for C1 and C5 and AP view for the other vessel segments.
3. Diameters (i.e. the contrast agent filled compartment of the vessel) are to be measured digitally in absolute values.
4. A ratio is to be built between all intradural and one extradural proportion (C5) of the vessel tree (as established by Weir and co-workers[2]).

[2] Weir B, Grace M, Hansen J, Rothberg C. Time course of vasospasm in man. *J Neurosurg*. 1978;48:173–178.

5. The ratios are to be used to analyse vessel diameters independently from the original magnification used. Angiographic vasospasm is defined as a ≥33% reduction in diameter in at least one vessel segment.

6. Angiographic vasospasm will be also assessed qualitatively as: none/mild (can be more than two major cerebral arteries [segments] with mild (less than 1/3 arterial narrowing) angiographic vasospasm and/or 1 segment with moderate angiographic vasospasm (1/3 to 2/3 arterial narrowing); moderate (at least 2 segments with moderate angiographic vasospasm and/or 1 or 2 segments with severe angiographic vasospasm [more than 2/3 arterial narrowing]); or severe (at least 3 segments with severe angiographic vasospasm). A segment corresponds to a major cerebral artery.

7. Additionally, a semiquantitative rating of the distal vessel segments according to this scheme will be performed.

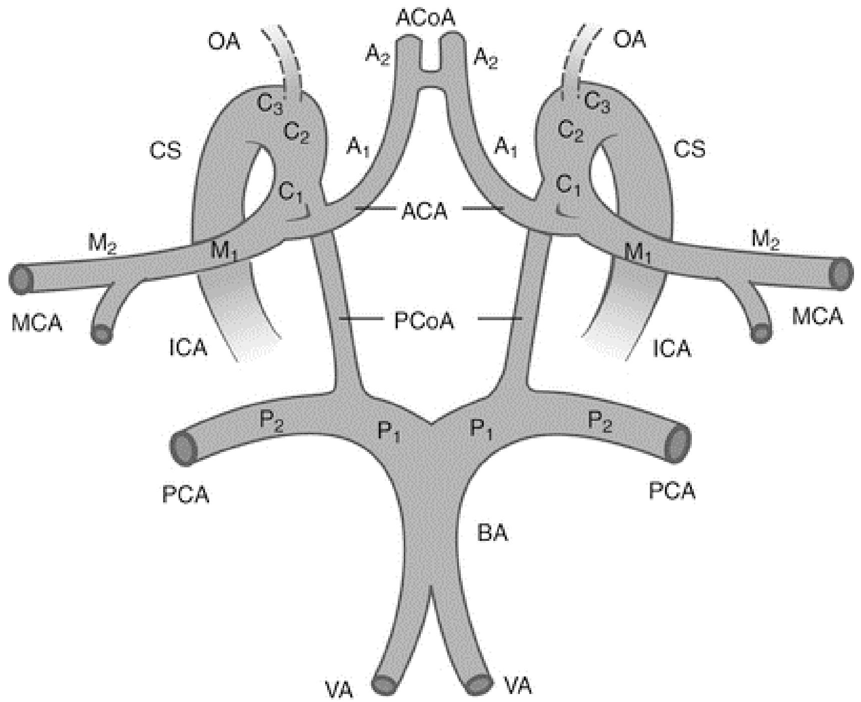

Nomenclature of the basal cerebral arteries of the circle of Willis. ACA, anterior cerebral artery (segments A1, A2); ACoA, anterior communicating artery; BA, basilar artery; CS, carotid siphon (segments C1-C3); ICA, internal carotid artery; MCA, middle cerebral artery (segments M1, M2); OA, ophthalmic artery; PCA, posterior cerebral artery (segments P1, P2); PCoA, posterior communicating artery; VA, vertebral artery.

All CT examinations will be assessed by a neuroradiologist blinded to the clinical data and treatment assignment. Infarct(s) on the CT brain scan performed 36 ± 12 hours postoperatively will be classified as surgery related and related to the respective vessel territory. New infarcts (compared to such postoperative CT) in the CT scan at visit 4 will be classified as vasospasm related and related to the respective vessel territory or to hemodynamic infarction.

Glasgow Coma Score (GCS)

The GCS is a standard measure to assess the level of consciousness of patients who have sustained head injuries. The GCS is part of standard management protocols and used for general decision-making for critically ill patients. It is an objective and reliable scale employed for initial and subsequent assessments. It consists of assessment of Eye responses, Verbal response, and Motor responses with each domain scoring a minimum of 1 to a maximum of 5, giving an overall score ranging from 3 (deep coma or death), to 15 (fully awake person).

Delayed Ischemic Neurological Deficit

Delayed Ischemic Neurological Deficit (DIND), also referred to as cerebral ischaemia, is defined according to the definition proposed by a multidisciplinary research group and reported on Stroke in 2010 [15. Vergouwen M.D., Vermeulen M., van Gijn J., Rinkel G.J., Wijdicks E.F., Muizelaar J.P., Mendelow A.D., Juvela S., Yonas H., Terbrugge K.G., Macdonald R.L., Diringer M.N., Broderick J.P., Dreier J.P., Roos Y.B. Definition of delayed cerebral ischemia after aneurysmal subarachnoid hemorrhage as an outcome event in clinical trials and observational studies: proposal of a multidisciplinary research group. Stroke. 2010 41(10):2391-5]. DIND is the occurrence of focal neurological symptoms impairment (such as hemiparesis, aphasia, apraxia, hemianopia, or neglect), or a decrease of at least 2 points on the GCS in awake patients (either on the total score or on one of its individual components [eye, motor on either side, verbal]). This should last for at least 1 hour, it is not apparent immediately after aneurysm occlusion, and cannot be attributed to other causes by means of clinical assessment, CT or MRI scanning of the brain, and appropriate laboratory studies.

Anti-vasospasm rescue therapy

Rescue therapy will be provided according to current guidelines for management of aSAH [1], and is defined as induced hypertension, super-selective intra-arterial infusion of vasodilator drugs, or balloon angioplasty.

Clinical Outcome

Modified Rankin Scale (mRS) (0)

The mRS is widely used as a functional outcome measure in stroke. The purpose of the Rankin Focused Assessment (RFA) is to assign patients to mRS grades in a systematic way. The assessment consists of sections corresponding to levels of disability among stroke survivors on the mRS.

Glasgow Outcome Scale (Extended) (GOSE) (0)

The Glasgow Outcome Scale is a global scale for functional outcome that rates patient status into one of five categories: Dead, Vegetative State, Severe Disability, Moderate Disability or Good Recovery. The Extended Glasgow Outcome Scale (GOSE) provides more detailed categorisation into eight categories by subdividing the categories of severe disability, moderate disability and good recovery into a lower and upper category.

Montreal Cognitive Assessment tool (MoCA) (0)

The Montreal Cognitive Assessment (MoCA) was designed as a rapid screening instrument for mild cognitive dysfunction. It assesses different cognitive domains: attention and concentration, executive functions, memory, language, visuoconstructional skills, conceptual

thinking, calculations, and orientation. The time to administer the MoCA is approximately 10 minutes. The total possible score is 30 points; a score of 26 or above is considered normal.

Safety Assessments

Vital Signs

Blood pressure and pulse rate will be measured automatically in supine position after the subject has rested comfortably for 3 minutes and in standing position after 1 minute (if possible) using an automated instrument. Respiration rate and body temperature will be measured in the supine position. Additional vital signs may be added for safety of the patients.

Hypotension is defined as mean systolic blood pressure <80 mm Hg, lasting over 15 minutes, and requiring treatment other than IV fluid resuscitation, and occurring any time after drug administration up to day 21.

12 Lead ECG

Computerised 12-lead ECG recordings will be obtained. Each lead shall be recorded for at least 3 beats at a speed of 25 mm/sec.

The following parameters will be recorded: rhythm, ventricular rate, PR interval, QRS duration, QT and QTc.

Bacterial Meningitis

Bacterial meningitis will be defined as organisms present on CSF culture If this is diagnosed, it will be treated with appropriate antibiotics by the treating physicians, with consultation from specialists in infectious diseases if deemed necessary.

Clinical Evaluations

Medical History

All patients will have medical history taken followed by thorough physical examination as part of their admission routine.

Medical history will include evaluation (past or present) of the following: general, head and neck, eyes, ears, nose throat, chest/respiratory, heart/cardiovascular, gastrointestinal/liver, urogenital, musculoskeletal/extremities, skin, neurological/psychiatric, endocrine/metabolic, haematologic/lymphatic, allergies/drug sensitivities, past surgeries, substance abuse or any other diseases or disorders.

Radiology

Incidence of moderate to severe cerebral angiographic vasospasm assessed by digital subtraction angiography (DSA) within at 8±1 days after aneurysm rupture where angiographic vasospasm is defined as a ≥33% reduction in diameter in at least one vessel segment by comparison to preoperative (pre NicaPlant® implantation) angiography and/or qualitatively assessed as moderate to severe vasospasm (in case the baseline measurement is missing). A day 8±1 angiogram will be performed even if the patient has no clinical or

sonographic evidence of vasospasm. If the patient develops clinical or sonographic changes suggestive of vasospasm prior to day 8, and angiogram will be performed to confirm the vasospasm and the angiographic measurement will replace the one scheduled at day 8±1. In case the patient develops clinical or sonographic changes suggestive of vasospasm after the day 8±1 DSA and prior to patient discharge, an angiogram will be performed to confirm the vasospasm, unless not clinically indicated. Definition has been reported in section 8.4.

Computed tomography angiography (CTA) has also been used to evaluate cerebral angiographic vasospasm in this patient population, however the sensitivity and specificity of the method does not directly compare to the DSA In order to avoid bias in assessments due to different frequency of use of DSA or CTA in the two patient groups, only DSA will be used in the current study to assess cerebral angiographic vasospasm. Patients who would have received a CTA will therefore have a higher exposition to radiation due to the DSA.

Additionally, a CT scan of the brain will be performed at screening, 36±12 hours post-surgery and at day 14±1, or before patient discharge, in order to identify new cerebral infarctions.

New cerebral infarction is defined as the presence of cerebral infarction on CT of the brain not present on the CT scan performed within 36±12 hours after aneurysm occlusion, and not attributable to other causes such as the surgical clipping procedure. Hypodensities on CT imaging resulting from ventricular catheter or intraparenchymal haematoma should not be regarded as cerebral infarction [15]. Magnetic resonance imaging has also been used to detect infarcts in this patient population, however the sensitivity and specificity of the method does not directly compare to the CT scan. In order to avoid bias in assessments due to different frequency of the use of CT versus MRI scan in the two patient groups, only CT scan will be used in the current study.

Transcranial Doppler ultrasonography (TCD) Recordings

TCD is scheduled once daily to reliably screen for the presence of vasospasm, that will then need to be confirmed by DSA.

Clinical utility of TCD is established for detection and monitoring of angiographic vasospasm in patients with aSAH (Type A, Class I-II) [16]. Vasospasm may be diagnosed as a mean flow velocity of >150 cm/s in at least one middle cerebral artery or by a rise in the flow velocities of > 50 cm/s from the previous TCD, or Lindegaard ratio (MCA velocity/ICA velocity) > 6. In this study, TCD is measured once daily from the day of bleed to day of discharge or day 21±1 post aneurysm. If the patient develops sonographic changes suggestive of vasospasm prior day 8, DSA will be performed to confirm vasospasm, this would replace the one scheduled for day 8. In case the patient develops sonographic changes suggestive of vasospasm after the day 8±1 DSA and prior to patient discharge or day 21, an angiogram will be performed to confirm the vasospasm, unless not clinically indicated.

TCDs will be performed by a blinded experienced member of the medical or neuro-intensive care team or other appropriately trained personnel who have otherwise carried out the same procedure on the same group of patients. The readings will be kept in the patient's medical notes and values will be entered on to the assessment CRF by the research team.

Risk Threshold and Degree of Distress

Clinical trials with patients lacking capacity must show a minimum of distress and other foreseeable risks for the patients. Consequently, the degree of distress and the risk threshold must be defined by the investigator and continuously checked for each patient.

The following table shows the risks and degree of distress of study specific measures which will be done additionally to standard procedures.

**Measure**	**Degree of distress**	**Risks**
Informed consent	minimal degree of distress	
Pregnancy test (urin or serum)	minimal degree of distress	In case of blood sampling: indisposition, vertigo, pain at the puncture location, haematoma at the puncture location<br>In rare instances, infection at the puncture location may occure.
ECG (every second day)	minimal degree of distress	No known risks
PK Plasma	minimal degree of distress	Indisposition, vertigo, pain at the puncture location, haematoma at the puncture location<br>In rare instances, infection at the puncture location may occure.
TCD (daily)	minimal degree of distress	No known risks (TCD is a non-invasive measure and is considered painless.)
Implantation of NicaPlant®	minimal degree of distress	The patient is anesthetised.

Minimal degree of distress is defined as: the expected discomforts for the patient are temporary and minor.

Clinical Laboratory Evaluations

The following investigations will be performed after the acute admission of the subject to the neurosurgical centre and following consent of the patient to the study. The details will be recorded on the CRF. Samples will be analysed at the local laboratory of the hospital:

Biochemistry

Enzymes	AST, ALT, Alkaline Phosphatase, Gamma-GT, LDH
Electrolytes	Sodium, Potassium, Calcium
Substrates	Creatinine, Total Bilirubin, Blood Urea Nitrogen
Coagulation	aPTT, INR

Haematology

Parameters: Hematocrit, Hemoglobin, Erythrocytes, MCV, MCHC, MCH, Total Leukocyte Count, Thrombocytes, CRP

Blood Sampling

During the inpatient stay, plasma samples for Pharmacokinetic, Haematology and serum biochemistry tests (FBC, U&Es, LFT and CRP) are taken at baseline and at the scheduled visits up to patient discharge or until day 21. Plasma samples for Pharmacokinetic are additionally taken when blood is drawn for medical reasons but only once per day.

Pregnancy Testing

Women of childbearing potential (all premenopausal women, or in cases where menstrual status cannot be ascertained i.e. women under the age of 55) should have a pregnancy test (urine or blood) performed at screening before implantation.

CSF Sampling

A number of patients will have an EVD drain placed for clinical reasons. These patients would normally, on clinical grounds, have regular CSF sampling ranging from daily to twice weekly.

In this study, in case CFS is removed for medical reason, approximately 500µl CSF will be taken until patient discharge, day 21 or the EVD drain is removed as per standard of care. The samples will be used to determine CSF nicardipine levels. In patients where is possible, on the day of clip ligation CSF sampling will be carried out at 0 hours (pre NicaPlant® administration). Only samples collected in patients treated with NicaPlant® will be analysed at the end of this study for the purposes of determining nicardipine levels. Total CSF taken for medical reasons will also be recorded.

Clinical Outcome

Clinical Outcome is measured at Weeks 12 & 52 post-aneurysm rupture by modified Rankin Scale, Glasgow Outcome Scale (Extended), Montreal Cognitive Assessment tool. Additionally, mortality caused by SAH complications will be recorded.

Non-Standard Assays or Procedures

Blood sampling will be carried out in all patients on day of clip ligation at 0, 6, 12 & 24 h post clipping (±20 minutes each) and on the scheduled visits after aneurysm rupture (time of sampling to be recorded) until patient discharge or day 21, whichever occurs first. Only samples collected in patients treated with NicaPlant® will be analysed at the end of this study for the purposes of determining nicardipine levels.

ADVERSE EVENT REPORTING AND HOSPITALISATION

Definitions

Adverse Event (AE): Any untoward medical occurrence in a patient administered a pharmaceutical product and that does not necessarily have a causal relationship with this treatment.

An adverse event can therefore be any unfavourable and unintended sign (including laboratory abnormal finding), system or disease temporally associated with the use of the medicinal product, whether or not considered as related to the investigational medicinal product.

Adverse Drug Reaction (ADR): Any untoward and unintended responses to an investigational medicinal product related to any dose administered.

All adverse events judged by either the reporting Investigator or the Sponsor as having a reasonable causal relationship to a medicinal product qualify as adverse reactions. The expression “reasonable causal relationship” means to convey in general that there are facts or evidence meant to suggest a causal relationship.

A Serious Adverse Event (SAE): Untoward medical occurrence or effect that at any dose falls in one or more of the following categories:

- Results in death
- Is life-threatening
- Requires hospitalisation or prolongation of existing inpatients’ hospitalisation. Hospitalisation refers to a situation whereby an AE is associated with unplanned overnight admission into hospital.
- Results in persistent or significant disability or incapacity
- Is a congenital anomaly or birth defect
- Is a medically significant adverse event

Expectedness

An expected adverse reaction is an adverse reaction, the nature or severity of which is consistent with the applicable product information (Investigator’s Brochure for an unapproved investigational product or Summary of Product Characteristics or approved Package Insert for an authorised product), otherwise it is considered unexpected.

Intensity of adverse event

Each adverse event must be rated in its severity according to the following:

Mild: A type of adverse event that is usually transient and may require only minimal treatment or therapeutic intervention. The event does not generally interfere with usual activities of daily living.

Moderate: A type of adverse event that is usually alleviated with additional specific therapeutic intervention. The event interferes with usual activities of daily living, causing discomfort, but poses no significant or permanent risk of harm to the subject.

<u>Severe:</u> Marked limitation in activity; medical intervention/therapy required, hospitalisation possible

<u>Life-threatening:</u> An event in which the patient was at risk of death at the time of the event. It does not refer to an event that hypothetically might have caused death, if it was more severe

Causality assessment

The investigator must assign causality to each adverse event in relation to the IMP based on the following definitions:

Not related:	AE, including laboratory test abnormality, with an incompatible time relationship to IMP administration, and that could be explained by underlying disease or other drugs or is incontrovertibly not related to the Investigational Product.
Unlikely Related	an AE, including laboratory test abnormality, with a reasonable time relationship to IMP administration which makes a causal relationship improbable, and in which other drugs, chemicals or underlying disease provide plausible explanations
Possibly related:	AE, including laboratory test abnormality, with a reasonable time relationship to IMP administration, but which also could be explained by concurrent disease or other medications.
Probably related:	AE, including laboratory test abnormality, with a reasonable time relationship to IMP administration that is unlikely to be attributed to concurrent disease or other medications.
Definitely related:	AE, including laboratory test abnormality, with plausible time relationship to IMP administration and which cannot be explained by concurrent disease or concomitant medications.

Action taken regarding the study drug

Typically, in studies involving IMP the action taken regarding study drug must be described in terms of changes to dose and/or frequency. As NicaPlant® is implanted once (at the time of aneurysmal clipping) it cannot be withdrawn.

Outcome

Each AE must be rated by selecting one of the following:

- Recovered / resolved
- Recovering / resolving
- Not recovered / not resolved
- Recovered with sequelae / resolved with sequelae
- Fatal

- Unknown

Recording adverse events

It is the responsibility of the investigator to collect all AEs (both serious and non-serious).

All AEs occurring during the study must be documented on the appropriate section of the case report form (CRF).

If an AE is considered serious, it must also be recorded and reported as a Serious Adverse Event Report.

AEs occurring after day 21±1 will be collected but will no longer be considered treatment emergent.

Reporting serious adverse events to the sponsor

All SAEs must be reported to NeuroScios PV within 24 hours of awareness, regardless of causal relationship. For SAE reporting, the part 11 CFR compliant safety database Aquila CTPV provided by NeuroScios GmbH will be used. For each trial site investigators will be provided with an access to the database on a dedicated server. SAEs will be signed electronically by the investigator within the database and will be automatically distributed to PV, sponsor representative(s), the project manager and the investigator as confirmation of receipt. Sponsor medical monitor/medical advisor is responsible for expectedness evaluation of the reported event.

Each SAE form must be printed by a member of the PV team and retained in the TMF in paper form. MedDRA coding of events will be performed by PV Unit within Aquila CTPV.

All SAEs occurring until 30 days after study end of each patient must be reported to by the Investigator or designated assistant is made aware of the event and by full report as soon as possible thereafter.

In case of system failure of Aquila, all SAEs must be completed in the corresponding paper version in the investigator site file (ISF) and emailed to: **safetybit@neuroscios.com** within 24 hours after knowledge of the event.

Pregnancies occurring during the study must be reported immediately by fax or email using the Pregnancy Report Form.

In case that an event is both serious and unexpected and a reasonable causal relationship to IMP administration is given, it constitutes a suspected unexpected serious adverse reaction. PV will report any SUSARs occurring in the trial to the relevant CAs, Ethics Committees (EC(s)), all study PIs and the DSMB as outlined in section 0.

NeuroScios will keep the Investigator and DSMB (section 0) informed of all SUSARs reported to them for the product under investigation, from anywhere in the world, for the duration of the trial at a frequency appropriate to the trial.

In addition, any new safety information that would adversely affect the safety of patients or the conduct of the trial will be reported by to the CAs, IECs, DSMB and Investigators. If the

trial is to be suspended as a result of a SUSAR, or due to any urgent safety measure taken, the CA and corresponding ECs will be notified as soon as possible and within three days of the decision.

BIT Pharma, with the assistance of NeuroScios GmbH, will submit a Development Safety Update Report to the CAs and ECs annually and/or additional tabular line listings of adverse events, if so requested.

Hospitalisation

The length of ICU stay and the length of hospital stay after aSAH will be recorded.

DATA MANAGEMENT

Data will be recorded on a CRF by the Investigator (or designee). The database, data entry and electronic checks will be developed using a Clinical Database Management System. Computerised data cleaning checks will be used in addition to manual review to check for discrepancies and to ensure consistency and completeness of the data. An electronic audit trail system will be used to track all data changes in the database.

A 100 % quality control check of the data entry will be performed on a randomly selected sample of the CRFs.

Medical history findings and adverse events will be coded using the MedDRA dictionary; medications will be coded using the World Health Organisation Drug dictionary.

A detailed Data Management Plan will be produced before study commencement.

STATISTICAL ANALYSIS PLAN

A detailed statistical analysis plan (SAP) will be produced after finalising the protocol.

Power & Sample size

In order to ensure that the investigation of efficacy is adequately powered, the sample size requirements are derived for the primary endpoint of interest.

The primary endpoint is the Incidence of Cerebral Angiographic Vasospasm (Moderate to Severe) at day 8 and these rates are compared for the active and control treatment groups using the Fishers Exact test for differences in proportions. PASS software (Version 12; NCSS LLC, Kaysville, UT, USA) was used for all calculations.

A total of 40 patients (20 in each group) is in fact sufficient to detect a clinically relevant difference of 50% between the active treatment and control group (active: 20%, control: 70%) with a two tailed significance level of 5% and 84% power.

The Incidence rates from previous published work (Barth et al.,2007) were used as an indicator of the likely incidence rates observed in a similar population with similar investigations (Active: 7%, Control: 73%).

Populations for analysis

Three main patient analysis sets will be described. These include the Full Analysis Set (FAS), Per protocol (PP) and Safety sets. The FAS set is considered the primary analysis set for all primary and secondary efficacy endpoints. The PP set will also be used to analyse the primary efficacy endpoint and will consist of those patients in the FAS set without any major protocol deviations. These will be defined in the SAP. The Safety set is considered the primary set for all safety evaluations.
For the purpose of evaluating PK and PD endpoints, separate Pharmacokinetic and Pharmacodynamic analysis sets will be defined.

All available data of deceased patients will be included in the analysis sets.

Statistical endpoints

Aim:

The objective is to assess the efficacy of NicaPlant® (active group) in comparison to routine Standard of care (control group). This will be based on statistical methods and be supported by clinical assessment of the relevance of the overall findings.

Null Hypothesis:

As this is an evaluation of the superiority of NicaPlant® over Standard of care. The Null hypothesis assumed throughout that there is no difference between the active and control groups for any of the assessments performed. The Alternative hypothesis is that the two groups do in fact differ.

A series of hypothesis tests and, where appropriate, supporting statistical models will be used to investigate these differences. Where possible, indicators of statistical significance (p values) will be quantified with estimates of the magnitude of the treatment differences and supporting the 95% confidence intervals.

Data Descriptions:

Data will be summarised according to patient population (FAS/PP/Safety), dose group (10 Implant/Control), assessment (Visit 1-6 / day where appropriate) and time (where appropriate). Data collected on visit 7 will be reported and analysed in an addendum to the study report.

Categorical variables will be summarised as proportions (with 95% Clopper-Pearson confidence intervals) and frequencies (if appropriate) and as odds ratios (with 95% Wald confidence intervals), where logistic regression models are used. Continuous variables will be summarised as Least square means and 95% confidence intervals, where analysis of variance

(ANOVA) models are used and then also as supporting descriptive statistics (n, mean, median, standard deviation, minimum, and maximum). Graphical displays appropriate to these types of data will be used to present important findings.

Treatment Group Comparability:

Surgical details (including Clip ligation & Implantation) and measures at baseline (including: demographics, medical history, concomitant medication, physical examination, pregnancy test) will be summarised descriptively by treatment group (10 Implant/Control) and overall. No formal comparisons of the treatment groups at baseline will be made.

Efficacy Endpoints:

The primary efficacy endpoint is the incidence of cerebral angiographic vasospasm (moderate or severe) at Day 8±1. This is considered a binary outcome and the difference between the 10 Implant and Control groups will be investigated using Fishers Exact test. Proportions in the two groups with 95% Clopper-Pearson confidence intervals will be used to quantify the magnitude of the difference. In case cerebral vasospasm (moderate or severe) is occurring before day 8, this measurement will replace the assessment at day 8±1.

A supporting Ordinal logistic regression model (utilising all classified categories) will also be used to provide odds ratio estimates (+/-95% confidence intervals) for the treatment effect and associated significance levels (p-values).

Secondary endpoints include the:

-Incidence of new cerebral infarcts on CT scan at day 14±1, or before patient discharge, (compared post-treatment CT scan).

Exploratory endpoints include the:

-Incidence of morbidity/mortality (DIND; death due to vasospasm, DIND, infarcts or complications due to anti-vasospasm therapy) by patient discharge.

-Need of anti-vasospasm rescue therapy by patient discharge.

-Clinical outcomes at week 12 and week 52, following assessment with modified Rankin Scale (mRS) scored 0-6 (with incidence of 'Good' outcome scores 0-2) and Glasgow Outcome Scale (extended) (GOSE) scored 0-8 (with incidence of 'Favourable' outcome scores 6-8), together with the Montreal Cognitive Assessment Tool (MOCA) (score 0-30 higher better) and Health related quality of life, assessed using the EQ-5D-5L and SF36. The Length of stay in hospital and the ICU will also be assessed as measures of clinical outcome .

The additional information reported as an addendum to the final report will specifically include the final assessment for clinical outcome (mRS and GOSE), assessed at week 52, together with any additional adverse events (specifically those that are CNS related), together with the MOCA, EQ-5D-5L and SF36.

These data can be considered binary, ordered categorical or interval in nature.

All binary outcomes will be evaluated using the same methods as those for the primary endpoint (Fishers exact test).

Ordered categorical data will be analysed using the Cochran-Mantel-Haenzel (CMH) test (with modified ridits). Ordinal logistic regression may be supplemented if other covariates of interest are identified (e.g. age, gender, WFNS).

Normally distributed Interval data will be analysed using an analysis of variance (ANOVA) model including treatment group as fixed effect and (if possible) baseline or other explanatory variables as covariates (e.g. age, gender, WFNS). Estimates of treatment effects and treatment differences will use LS means and 95% confidence intervals.

Non-parametric alternatives, however, will be substituted if these data are non-normally distributed (eg: Length of stay). This may take the form of the Wilcoxon rank sum test or a ranked based ANOVA model.

Safety Endpoints:

All safety assessments performed including adverse events (and separately shunt-dependent hydrocephalus, bacterial meningitis), vital signs (blood pressure, pulse rate, respiratory rate and body temperature), ECG, haematology and biochemistry will be descriptively summarised. No formal analysis is intended.

Pharmacokinetic (PK) Endpoints:

Samples taken for drug concentration estimation in plasma and CSF are obtained post-surgery. Single plasma samples are obtained on day 8, 14 and 21 and on additional days when blood samples are taken for medical reasons but only once per day with single CSF samples obtained when feasible in patient provided for medical reasons with external ventricular drain at each occurrence when the CSF is removed for medical reasons and up to day of discharge or day 21.

Multiple plasma samples are obtained on Implantation day at 0, 6, 12 and 24h post implant (±20 minutes each) for evaluation of day 1 kinetics.

Plasma and CSF samples will be used to determine the total exposure to drug (AUC for multiple plasma samples and average concentration (Cav) for single CSF samples if sufficient CSF data is obtained).

a. Drug Profiles:

Plasma - Individual patient plasma drug concentration profiles over time for the active treatment group (10 Implant) will be presented graphically and in summary tables. These will identify the dose group (10 Implant) and assessment day (day 1, 8, 14, 21 and additional samples where appropriate) and where applicable sampling time (day 1: 0, 6, 12 and 24 hours).

Overall mean profile summaries for plasma will be produced according to dose group (10 Implant) and assessment day and time (where appropriate)for the relevant analysis set using descriptive statistics (n, arithmetic mean, SD, min, median, max, geometric mean, and coefficient of variation (CV)) and these will also be presented graphically using geometric means with linear and semi-logarithmic scales.

CSF – Individual patient CSF values will be listed by day, date and time and plotted individually over time as appropriate.

However, these values will only be descriptively and graphically summarised according to the active treatment group (10 Implant) by day/visit if, prior to database lock, there is considered sufficient data to present them in the final statistical and clinical study reports.

b. PK Parameters:

Plasma - The individual drug profiles will then be used to derive the respective PK curve parameters for each patient. This will involve non-compartmental methods.

The overall mean PK parameter summaries for plasma (defined in Section 2.4) and will then also be produced according to dose group (10 Implant) for the relevant analysis set, using descriptive statistics (n, arithmetic mean, SD, min, median, max, geometric mean and CV). Plasma PK parameters will be presented for both Day 1 kinetics (0-24 hours) and Day 1 – 21 whenever sufficient data is available. The $AUC_{0-\infty,}$ AUC_{0-t} and C_{max} plus other parameters defined in Section 2.4 for plasma will also be presented graphically.

Individual patient plasma PK parameter values will also be presented with the summary tables.

CSF – The C_{av} (Day 1 – 21) for CSF will be similarly presented to the plasma PK parameters if, prior to database lock, there is considered sufficient CSF data to present the C_{av} in the final statistical and clinical study reports. Individual patient C_{av} parameter values will be presented if sufficient data is available.

Pharmacodynamics Endpoints:

All PD endpoints will be summarised according to treatment group (10 Implant) and assessment day using descriptive statistics. Derived PD parameters will be similarly described.

The relationship between drug exposure and pharmacodynamic effect will be explored on an individual patient by patient basis, using the pharmacokinetic drug concentration over time profiles as well as pharmacokinetic exposure parameters (Plasma AUC_{0-t}, $AUC_{0-\infty}$ / CSF Day 1-21: Cav).

In addition, the influence of exposure on safety (occurrence of specific adverse events) and efficacy measures (vasospasm) may also be investigated.

ETHICS COMMITTEE / IRB APPROVAL

The study proposal will be submitted to the Independent Ethics Committee (IEC) in accordance with the national requirements.

The IEC shall give its opinion in writing before the clinical trial commences. The investigator should provide written reports to the IEC annually or more frequently if requested on any changes significantly affecting the conduct of the trial and / or increasing risk to the subjects.

REGULATORY REQUIREMENTS

The study will be authorised by the competent authority in each participating country.

Enrolment of subjects will not start until approval has been received from both the Ethics Committee(s) and Competent authorities.

The study will be conducted in accordance with the Declaration of Helsinki, Good Clinical Practice (GCP) and all other national requirements.

INFORMED CONSENT

The statutory requirements for informed consent of participants in clinical trials of investigational medicinal products (CTIMPs) are laid out in the European Clinical Trials Directive (EC2001/20).

Due to the nature of aSAH, it is expected that the majority of participants will be considered to be 'incapacitated adults' at the time of entry into the study, where an incapacitated adult is defined as "an adult unable by virtue of physical or mental incapacity to give informed consent". Overall the informed consent procedure shall not cause any delay in the treatment or reduce the safety of the patients.

a. Informed Consent procedure in Austria:

The hierarchy for consent is considered to be:

1. Patients with Capacity<br>Those patients able to give written informed consent
2. Personal Legal Representative<br>A person not connected with the conduct of the trial who is:<br>(a) Suitable to act as the legal representative by virtue of their relationship with the adult,<br>and<br>(b) Available and willing to do so
3. No representative<br>In emergency situations where the treatment to be given to an incapacitated adult as part of the trial needs to be given urgently, time may not allow for the written consent of a legal representative to be obtained first.<br>Written informed consent will be obtained as soon as the participant is responsive or a representative is available.

Patients with capacity

Written and verbal versions of the patient information and informed consent form will be presented to the participants detailing the exact nature of the trial, what it will involve for the subject, the implications and constraints of the protocol and the known side effects and risks involved in taking part. It will clearly state that the participant is free to withdraw from the trial at any time for any reason without prejudice to future care and with no obligation to give a reason for withdrawal.

The patients will be allowed as much time as they need to decide whether to participate in the trial. The 48-hour timeline for implantation will not be used to put pressure on the patient to make a decision.

The Investigator should explain to the patient that they are at liberty to refuse entry to the trial or, should they decide to participate, to withdraw from the trial at any time. Such a decision will not, in any way, affect their future management or treatment.

Written informed consent will be obtained by means of participant dated signature with dated signature of the person who presented and obtained the informed consent. The person who obtains the consent must be suitably qualified and experienced and have been authorised to do so by the Coordinating/Principal Investigator and the delegation log completed to document this. A copy of the signed informed consent will be given to the participant and a copy will be kept in the patient’s notes. The original signed form will be retained in the Investigator Site File (ISF).

Patients lacking capacity with Personal Legal Representative immediately available

Where patients lack capacity and the Personal Legal Representative is immediately available in person, an identical approach will be taken substituting the Personal Legal Representative for the patient.

For patients that were unconscious at screening, and informed consent was obtained from their Personal Legal Representative, written informed consent will be obtained and documented from the patient as soon as they regain consciousness sufficiently to do so, respecting their right to withdraw from the study should they wish to.

The investigator will attempt to obtain consent until the completion of the study.

Patients lacking capacity, with no personal legal representative may be recruited into the study in emergency situations where the treatment to be given to an incapacitated adult as part of the trial needs to be given urgently, time may not allow for the written consent of a legal representative to be obtained first.

Written consent will be obtained as soon as the participant is responsive or a representative is available.

b. Informed Consent procedure in Germany:

Patients lacking capacity may be recruited into the study if an independent physician, who is not involved in the study and is able to assess the situation, confirms that the presumed will of the patient has been determined and respected. The independent physician must not be part of the study team and he has to be a medical specialist.

Determination procedure of the presumed will of the patient

Patients lacking capacity will only be included in this clinical trial if their participation is in accordance with their presumed will. Patients' presumed will, will be, in general, to participate in the clinical trial because they will have the chance to receive a potentially superior treatment to the standard therapy. Patients not randomised to the treatment arm will receive the standard therapy.

The investigator will carefully check if patients have ever expressed that they are against participating in clinical trials, even if the trial is in their best interest. To determine this, the patient's companions, if available, will be consulted. Even if patients are only partially able to follow the patient information because of aSAH's symptomatology, they are to be informed and if they do not wish to participate in the clinical trial, they will not be included.

The determination of the presumed will of the patient is the duty of the investigator. The investigator must consider all known indications to determine the presumed will of the patient. To prevent the investigator and the patient from wrong assessments, an independent physician, who is not involved in the study, must be consulted.

The procedure of determining the presumed will of the patient is to be documented. As soon as the patient is sufficiently recovered, written informed consent will be obtained. If they wish to withdraw from the study, their wishes will be respected.

The investigator will attempt to obtain consent until the completion of the study.

DIRECT ACCESS TO SOURCE DOCUMENTATION / DATA

The investigator must permit trial-related monitoring, audits by Sponsor, Sponsor representative or regulatory authority, Ethics Committee review or regulatory inspection, providing direct access to source data / documents.

The CRF is defined as source data for rating scales i.e. Glasgow Coma Scale.

STUDY MONITORING

It is understood that the study monitor(s) will contact and visit the investigator/clinical site before the study, regularly throughout the study and after the study has been completed. At these visits, the monitor(s) will inspect various study records; case report forms, investigator site file and source data (patient records). No information in these records about the identity of the subjects will leave the Trial site. The sponsor will maintain confidentiality of all subject records. The investigator and / or site staff will be expected to be available if requested by the monitor.

QUALITY ASSURANCE

The sponsor (Brain Implant Therapeutics Pharma GmbH) or representative may perform an audit at any time according to the sponsor's Standard Operating Procedure (SOP), in order to verify whether the study is being conducted according to GCP.

INSURANCE

Appropriate insurance cover has been undertaken in favour of patients participating in clinical trials. The cover is provided to the patient on terms and conditions of the clinical trial insurance. Insurance cover exists for health damages as a result of measures carried out in connection with the clinical trial.

CONFIDENTIALITY

All study documents are provided by the sponsor in confidence to the investigator and appointed staff. No study material may be disclosed to any party not directly involved in the study without written permission from the sponsor.

The investigator must assure that subject's anonymity will be provided. The investigator will keep a separate list with at least the initials, the subject's study number, names, addresses and telephone numbers. The investigator will maintain this for as long as requested by the sponsor.

PREMATURE TERMINATION OF THE STUDY

Both the sponsor and the investigator reserve the right to terminate the study at any time. Should this be necessary, the procedures for an early termination or temporary halt will be arranged after consultation with all parties.

RECORD RETENTION

After completion of the study, all documents and data relating to the study will be kept in an orderly manner by the Investigator in a secure study file.

Essential documents must be retained for at least two years after the final marketing approval in an ICH region or until two years have elapsed since the formal interruption of the clinical development of the product under study.

It is the responsibility of NeuroScios GmbH to inform the investigator of when these documents can be destroyed. The Investigator must contact NeuroScios GmbH or the sponsor before destroying any trial-related documentation. In addition, all subjects' medical records and other source documentation will be kept for the maximum time permitted by the institution.

PUBLICATION OF RESULTS

The sponsor is entitled to publish and/or present any results at scientific meetings, and to submit clinical trial data to national and international Regulatory Authorities. The sponsor reserves the right to use such data for industrial purposes.

Investigators must inform the sponsor before using the results of the study for publication or conference and internal presentation and agree to provide the sponsor with a copy of the proposed presentation.

DATA SAFETY MONITORING BOARD (DSMB)

A committee will be set-up to monitor safety throughout the trial period. The committee will comprise a group of at least three experts with the appropriate expertise - all of whom will be independent of the sponsor and will not be involved in the conduct of the trial.

A charter describing how the DSMB works and how it communicates with other study participants (e.g. steering committee), will be prepared.

The DSMB will review unblinded study information which will include:

- List of any protocol violations
- Numbers of patient withdrawals/reason for withdrawal
- Adverse/serious adverse events
- Laboratory data

Following review of the safety data, the committee will prepare a written blinded report, which will be forwarded to the steering committee and provide recommendations regarding modifications, continuation or termination of the study.

Where changes in the study conduct are recommended to the steering committee, sufficient (blinded) information will be provided to allow the sponsor to decide whether and how to implement these recommendations.

DSMB Meetings

The DSMB will convene under the following circumstances:

- 20 patients have been recruited and monitored to discharge or day 21, whichever occurs first.

- The DSMB must meet as soon as there has been a SUSAR.

In case of no SUSAR, the recruitment will continue throughout the DSMB meeting scheduled after 20 patients. Specifically, the recruitment will continue also during the time the DSMB is assessing the safety data in the initial 20 patients.

Interim Meetings

In case of a SUSAR occurrence during the trial, the DSMB will be informed by NeuroScios within 24h after expectedness assessment of event and an interim meeting will be scheduled.

Study Stopping Rules

The clinical investigation can be placed on hold / stopped early for two reasons and will be based on clinical judgement:

- The DSMB will consider recommending that the study is placed on hold or stopped if the adverse events associated with participation in the study are considered unacceptable.
- The DSMB will consider recommending that the study is placed on hold or stopped if the adverse events associated with NicaPlant®, in their opinion, significantly outnumber (in frequency or intensity) the adverse events associated with the normal standard of care.

STEERING COMMITTEE

The steering committee for the NicaPlant® clinical programme will be called the BIT-002 Executive Committee. The executive committee (which is blinded) will comprise at a minimum, representatives of the sponsor and a Medical Advisor and representatives of NeuroScios; the committee will receive and review the reports from the DSMB, and take action as appropriate. This may be a decision to either continue, modify, or terminate the study.

REFERENCES

1. De Roij, N.K., Linn, F.H.H., van der Plas, J.A., Algra, A., and Rinkel, G.J.E. Incidence of subarachnoid haemorrhage: a systematic review with emphasis on region, age, gender and time trends. J. Neurol. Neurosurg. Psychiatry. 2007; 78:1365-1372.

2. Nieuwkamp, D.J., Setz, L.E., Algra, A., Linn, F.H.H., de Rooij, N.K., and Rinkel G.J.E. Changes in case fatality of aneurysmal subarachnoid haemorrhage over time, according to age, sex, and region: a meta-analysis. Lancet Neurol. 2009; 8: 635-42.

3. Kreiter, K.T., Copeland, D., Bernardini, G.L., Bates, J.E., Peery, S., Claassen, J., Du, E., Stern, Y., Connolly, E.S., and Mayer, S.A. Predictors of cognitive dysfunction after subarachnoid haemorrhage. Stroke. 2002; 33: 200–8.

4. Pickard J.D., Murray, M.G., Illingworth, R., Shaw, M.D.M., Teasdale, G.M., Foy, P.M., Humphrey, P.R.D., Lang, D.A., Nelson, R., Richards, P., Sinar, J., Bailey, S., and Skene, A. Effect of oral nimodipine on cerebral infarction and outcome after subarachnoid haemorrhage: British aneurysm nimodipine trial. BMJ 1989 11; 298: 636-42.

5. Dorhout Mees, S.M., Rinkel, G.J., Feigin, V.L., Algra, A., van den Bergh, W.M., Vermeulen, M., et al. Calcium antagonists for aneurysmal subarachnoid haemorrhage. Cochrane Database Syst Rev. Issue 4, 2008: CD000277

6. Langham, J., Reeves, B.C., Lindsay, K.W., van der Meulen, J.H., Kirkpatrick, P.J., Gholkar, A.R., Molyneux, A.J., Shaw, D.M., Copley, L., Browne, J.P. Steering Group for National Study of Subarachnoid Haemorrhage, Variation in outcome after abuarachnoid haemorrhage: a study of neurosurgical untis in UK and Ireland. Stroke. 2009; 40(1): 111-8.

7. Connolly, E.S. Jr.,, Rabinstein, A.A., Carhuapoma, J.R., et al. Guidelines for the management of aneurysmal subarachnoid haemorrhage: a guideline for healthcare professionals from the American Heart Association/American Stroke Association. Stroke. 2012; 43(6): 1711–37.

8. Steiner, T., Juvela, S., Unterberg, A., Jung, C., Forsting, M., and Rinkel, G. European Stroke Organization Guidelines for the Management of Intracranial Aneurysms and Subarachnoid Haemorrhage. Cerebrovascular Disease. 2013; 35, 93-112.

9. Kasuya, H., Onda, H., Takeshita, M., Okada, Y., and Hori, T. Efficacy and safety of nicardipine prolonged-release implants for preventing vasospasm in humans. Stroke, 2002, 33, 1011-1015.

10. Kasuya, H., Onda, H., Sasahara, A., Takeshita, M., and Hori, T. Application of nicardipine prolonged-release implants: Analysis of 97 consecutive patients with acute subarachnoid haemorrhage. Neurosurgery, 2005, 56, 895-905.

11. Barth, M., Capelle, H.H., Weidauer, S., Weiss, C., Münch, E., Thomé, C., Luecke, T., Schmiedek, P., Kasuya, H., and Vajkoczy, P. Effect of nicardipine prolonged-release implants on cerebral vasospasm and clinical outcome following severe aneurysmal

subarachnoid haemorrhage – a prospective, randomised, double-blind phase IIa study. Stroke, 2007, 38, 330-336.

12. Krischek, B.; Kasuya, H.; Onda, H.; Hori, H. Clinical trial of nicardipine prolonged-release implants for preventing vasospasm: Analysis of 100 consecutive patients. Neurol. Med. Chir. (Tokyo), 2007, 47, 389-396.

13. Kasuya, H. Clinical trial of nicardipine prolonged-release implants for preventing cerebral vasospasm: multicentre cooperative study in Tokyo. Acta Neurochir Suppl. 2011;110(2):165–7.

14. Hänggi, D., Etminan, N., Macdonald, R.L., Steiger, H.J., Mayer, S.A., Aldrich, F., Diringer M.N., Hoh, B.L., Mocco, J., Strange, P., Faleck, H.J., and Miller, M. NEWTON: Nimodipine Microparticles to Enhance Recovery While Reducing Toxicity After Subarachnoid Haemorrhage. (2015) Neurocrit Care. 2015 Oct; 23(2):274-84.

15. Vergouwen M.D., Vermeulen M., van Gijn J., Rinkel G.J., Wijdicks E.F., Muizelaar J.P., Mendelow A.D., Juvela S., Yonas H., Terbrugge K.G., Macdonald R.L., Diringer M.N., Broderick J.P., Dreier J.P., Roos Y.B. Definition of delayed cerebral ischemia after aneurysmal subarachnoid haemorrhage as an outcome event in clinical trials and observational studies: proposal of a multidisciplinary research group. Stroke. 2010 41(10):2391-5.

16. Slone MA, Alexandrov AV, Tegeler CH, Spencer MP, Caplan LR, Feldmann E, Wechsler LR, Newell DW, Gomez CR, Babikian VL, Lefkowitz D, Goldman RS, Armon C, Hsu CY, Goodin DS. Assessment: transcranial Doppler ultersonography: report of the Therapeutics and Technology Assessment Subcommittee of the American Academy of Neurology. Neurology. 2004 May 11;62(9):1468-81.

APPENDICES

Appendix 1 – Declaration of Helsinki

Recommendations Guiding Medical Physicians in Biomedical Research Involving Human Volunteers

Adopted by the 18th World Medical Assembly, Helsinki, Finland, June 1964 amended by the 29th World Medical Assembly, Tokyo, Japan, October 1975 and the 35th World Medical Assembly, Venice, Italy, October 1983 and revised 41st World Medical Assembly Hong Kong, 1989 and by the 48th World Medical Assembly, South Africa, October 1996

<u>Introduction</u>

It is the mission of the physician to safeguard the health of the people. His or her knowledge and conscience are dedicated to the fulfilment of this mission.

The Declaration of Geneva of the World Medical Association binds the physician with the words, "The health of my patient will be my first consideration", and the International Code of Medical Ethics declares that, "A physician shall act only in the patient's interest when providing medical care which might have the effect of weakening the physical and mental condition of the patient".

The purpose of biomedical research involving human volunteers must be to improve diagnostic, therapeutic and prophylactic procedures and the understanding of the aetiology and pathogenesis of disease.

In current medical practice most diagnostic, therapeutic or prophylactic procedures involve hazards. This applies especially to biomedical research.

Medical progress is based on research which ultimately must rest in part on experimentation involving human volunteers.

In the field of biomedical research a fundamental distinction must be recognised between medical research in which the aim is essentially diagnostic or therapeutic for a patient, and medical research, the essential object of which is purely scientific and without implying direct diagnostic or therapeutic value to the person volunteered to the research.

Special caution must be exercised in the conduct of research which may affect the environment, and the welfare of animals used for research must be respected.

Because it is essential that the results of laboratory experiments be applied to human beings to further scientific knowledge and to help suffering humanity, the World Medical Association has prepared the following recommendations as a guide to every physician in biomedical research involving human volunteers. They should be kept under review in the future.

It must be stressed that the standards as drafted are only a guide to physicians all over the world. Physicians are not relieved from criminal, civil and ethical responsibilities under the laws of their own countries.

I Basic Principles

Biomedical research involving human volunteers must conform to generally accepted scientific principles and should be based on adequately performed laboratory and animal experimentation and on a thorough knowledge of the scientific literature.

The design and performance of each experimental procedure involving human volunteers should be clearly formulated in an experimental protocol which should be transmitted for consideration, comment and guidance to a specially appointed committee independent of the investigator and the sponsor provided that this independent committee is in conformity with the laws and regulations of the country in which the research experiment is performed.

Biomedical research on human volunteers should be conducted only by scientifically qualified persons and under the supervision of a clinically competent medical person. The responsibility for the human volunteer must always rest with a medically qualified person and never rest on the volunteer of the research, even though the volunteer has given his or her consent.

Biomedical research involving human volunteers cannot legitimately be carried out unless the importance of the objective is in proportion to the inherent risk to the volunteer.

Every biomedical research project involving human volunteers should be preceded by careful assessment of predictable risks in comparison with foreseeable benefits to the volunteer or to others. Concern for the interest of the volunteer must always prevail over the interests of science and society.

The right of the research volunteer to safeguard his or her integrity must always be respected. Every precaution should be taken to respect the privacy of the volunteer and to minimise the impact of the study on the volunteer's physical and mental integrity and on the personality of the volunteer.

Physicians should abstain from engaging in research projects involving human volunteers unless they are satisfied that the hazards involved are believed to be predictable. Physicians should cease any investigation if the hazards are found to outweigh the potential benefits.

In publication of the results of his or her research, the physician is obliged to preserve the accuracy of the results. Reports on experimentation not in accordance with the principles laid down in this Declaration should not be accepted for publication.

In any research on human beings, each potential volunteer must be adequately informed of the aims, methods, anticipated benefits and potential hazards of the study and the discomfort it may entail. He or she should be informed that he or she is at liberty to abstain from participation in the study and that he or she is free to withdraw his or her consent to participation at any time. The physician should then obtain the volunteer's freely-given informed consent, preferably in writing.

When obtaining informed consent for the research project the physician should be particularly cautious if the volunteer is in a dependent relationship to him or her or may consent under duress. In that case the informed consent should be obtained by a physician

who is not engaged in the investigation and who is completely independent of this official relationship.

In the case of legal incompetence, informed consent should be obtained from the legal guardian in accordance with national legislation. Where physical or mental incapacity makes it impossible to obtain informed consent, or when the volunteer is a minor, permission from the responsible relative replaces that of the volunteer in accordance with national legislation.

Whenever the minor child is in fact able to give consent, the minor's consent must be obtained in addition to the consent of the minor's legal guardian.

The research protocol should always contain a statement of the ethical considerations involved and should indicate that the principles enunciated in the present Declaration are complied with.

II Medical Research Combined With Professional Care (CLINICAL RESEARCH)

In the treatment of the sick person, the physician must be free to use a new diagnostic and therapeutic measure, if in his or her judgement it offers hope of saving life, re-establishing health or alleviating suffering.

The potential benefits, hazards and discomfort of a new method should be weighed against the advantages of the best current diagnostic and therapeutic methods.

In any medical study, every patient - including those of a control group, if any - should be assured of the best proven diagnostic and therapeutic method. This does not exclude the use of inert placebo in studies where no proven diagnostic or therapeutic method exists.

The refusal of the patient to participate in a study must never interfere with the physician-patient relationship.

If the physician considers it essential not to obtain informed consent, the specific reasons for this proposal should be stated in the experimental protocol for transmission to the independent committee.

The physician can combine medical research with professional care, the objective being the acquisition of new medical knowledge, only to the extent that medical research is justified by its potential diagnostic or therapeutic value for the patient

III Non-Therapeutic Biomedical Research Involving Human Volunteers (NON-CLINICAL BIOMEDICAL RESEARCH)

In the purely scientific application of medical research carried out on a human being, it is the duty of the physician to remain the protector of the life and health of that person on whom biomedical research is being carried out.

The volunteer should be volunteers - either healthy persons or patients for whom the experimental design is not related to the patient's illness.

The investigator or the investigating team should discontinue the research if in his/her or their judgement it may, if continued, be harmful to the individual.

In research on man, the interest of science and society should never take precedence over considerations related to the well-being of the volunteer.

.

1627 **Appendix 2 - Modified Rankin Scale (mRS)**

MODIFIED RANKIN SCALE (MRS)

Patient Name: ____________________________

Rater Name: ____________________________

Date: ____________________________

Score	Description
0	No symptoms at all
1	No significant disability despite symptoms; able to carry out all usual duties and activities
2	Slight disability; unable to carry out all previous activities, but able to look after own affairs without assistance
3	Moderate disability; requiring some help, but able to walk without assistance
4	Moderately severe disability; unable to walk without assistance and unable to attend to own bodily needs without assistance
5	Severe disability; bedridden, incontinent and requiring constant nursing care and attention
6	Dead

TOTAL (0–6):

References

Rankin J. "Cerebral vascular accidents in patients over the age of 60." *Scott Med J* 1957;2:200-15

Bonita R, Beaglehole R. "Modification of Rankin Scale: Recovery of motor function after stroke." ***Stroke* 1988 Dec;19(12):1497-1500**

Van Swieten JC, Koudstaal PJ, Visser MC, Schouten HJ, van Gijn J. "Interobserver agreement for the assessment of handicap in stroke patients." ***Stroke* 1988;19(5):604-7**

Appendix 3 - Glasgow Outcome Scale Extended (GOSE)

Glasgow Outcome Scale

The Glasgow Outcome Scale (GOS) is a global scale for functional outcome that rates patient status into one of five categories: Dead, Vegetative State, Severe Disability, Moderate Disability or Good Recovery. The Extended GOS (GOSE) provides more detailed categorization into eight categories by subdividing the categories of severe disability, moderate disability and good recovery into a lower and upper category:

Table 1: Extended Glasgow Outcome Scale (GOSE)

1	Death	D
2	Vegetative state	VS
3	Lower severe disability	SD -
4	Upper severe disability	SD +
5	Lower moderate disability	MD -
6	Upper moderate disability	MD +
7	Lower good recovery	GR -
8	Upper good recovery	GR +

Use of the structured interview is recommended to facilitate consistency in ratings.

References:

Jennett B, Bond M: Assessment of outcome after severe brain damage. Lancet 1:480–484, 1975.

Teasdale GM, Pettigrew LE, Wilson JT, Murray G, Jennett B. Analyzing outcome of treatment of severe head injury: A review and update on advancing the use of the Glasgow Outcome Scale. *Journal of Neurotrauma* 1998;15:587-597.

Wilson JTL, Pettigrew LEL, Teasdale GM. Structured interviews for the Glasgow Outcome Scale and the Extended Glasgow Outcome Scale: Guidelines for Their Use. J Neurotrauma 15(8): 573-85. 1997.

Wilson JT, Slieker FJ, Legrand V, Murray G, Stocchetti N, Maas AI. Observer variation in the assessment of outcome in traumatic brain injury: experience from a multicenter, international randomized clinical trial. Neurosurgery. Jul;61(1):123-8; discussion 128-9. 2007 .

GOSE Page 1

POST DISCHARGE
STRUCTURED INTERVIEW FOR GOSE

Respondent: ☐ 0 = Patient alone 1 = Relative/friend/caretaker alone 2 = Patient plus relative/friend/caretaker

Conciousness:

1. Is the head-injured person able to obey simple commands or say any words?

○ Yes ○ No (VS)

Note: anyone who shows the ability to obey even simple commands or utter any word or communicate specifically in any other way is no longer considered to be in vegetative state. Eye movements are not reliable evidence of meaningful responsiveness. Corroborate with nursing staff and/or other caretakers. Confirmation of VS requires full assessment.

Independence at home:

2a. Is the assistance of another person at home essential every day for some activities of daily living?

○ Yes ○ No (VS) **If no: go to 3**

Note: for a NO answer they should be able to look after themselves at home for 24 hours if necessary, though they need not actually look after themselves. Independence includes the ability to plan for and carry out the following activities: getting washed, putting on clean clothes without prompting, preparing food for themselves, dealing with callers and handling minor domestic crises. The person should be able to carry out activities without needing prompting or reminding and should be capable of being left alone overnight.

2b. Do they need frequent help of someone to be around at home most of the time?

○ Yes (lower SD) ○ No (upper SD)

Note: for a NO answer they should be able to look after themselves at home up to eight hours during the day if necessary, though they need not actually look after themselves

2c. Was the patient independent at home before the injury?

○ Yes ○ No

Independence outside home:

3a. Are they able to shop without assistance?

○ Yes ○ No (upper SD)

Note: this includes being able to plan what to buy, take care of money themselves and behave appropriately in public. They need not normally shop, but must be able to do so.

3b. Were they able to shop without assistance before?

○ Yes ○ No

4a. Are they able to travel locally without assistance?

○ Yes ○ No (upper SD)

Note: they may drive or use public transport to get around. Ability to use a taxi is sufficient, provided the person can phone for it themselves and instruct the driver.

4b. Were they able to travel locally without assistance before the injury?

○ Yes ○ No

Work:

5a. Are they currently able to work (or look after others at home) to their previous capacity?

○ Yes **If yes, go to 6** ○ No

5b. How restricted are they?

a. Reduced work capacity? ○ a. (Upper MD)

b. Able to work only in a sheltered workshop or non-competitive job or currently unable to work? ○ b. (Lower MD)

1630 GOSE Page 3

5c. Does the level of restriction represent a change in respect to the pre-trauma situation?

○ Yes ○ No

Social and Leisure activities:

6a. Are they able to resume regular social and leisure activities outside home?

○ Yes **If yes, go to 7** ○ No

Note: they need not have resumed all their previous leisure activities, but should not be prevented by physical or mental impairment. If they have stopped the majority of activities because of loss of interest or motivation, then this is also considered a disability.

6b. What is the extent of restriction on their social and leisure activities?

- a. Participate a bit less: at least half as often as before injury ○ a. (Lower GR)
- b. Participate much less: less than half as often ○ b. (Upper MD)
- c. Unable to participate: rarely, if ever, take part ○ c. (Lower MD)

6c. Does the extent of restriction in regular social and leisure activities outside home represent a change in respect or pre-trauma

○ Yes ○ No

Family and friendships:

7a. Has there been family or friendship disruption due to psychological problems?

○ Yes ○ No **If no, go to 8**

Note: typical post-traumatic personality changes are: quick temper, irritability, anxiety, insensitivity to others, mood swings, depression and unreasonable or childish behaviour.

7b. What has been the extent of disruption or strain?

- a. Occasional - less than weekly ○ a. (Lower GR)
- b. Frequent - once a week or more, but not tolerable ○ b. (Upper MD)
- c. Constant - daily and intolerable ○ c. (Lower MD)

7c. Does the level of disruption or strain represent a change in respect to pre-trauma situation?

○ Yes ○ No

Note: if there were some problems before injury, but these have become markedly worse since the injury then answer yes to question [illegible]

Return to normal life:

8a. Are there any other current problems relating to the injury which affect daily life?

○ Yes (Lower GR) ○ No (Upper GR)

Note: other typical problems reported after head injury: headaches, dizziness, sensitivity to noise or light, slowness, memory failures and concentration problems.

8b. If similar problems were present before the injury, have these become markedly worse?

○ Yes ○ No

9. What is the most important factor in outcome?

○ a. Effects of head injury
○ b. Effects of illness or injury to another part of the body
○ c. A mixture of these

Note: extended GOS grades are shown beside responses on the CRF. The overall rating is based on the lowest outcome category indicated.
Areas in which there has been no change with respect to the pre-trauma situation are ignored when the overall rating is made

1631 GOSE Page 4

1632 **Appendix 4 - Montreal Cognitive Assessment tool (MoCA)**

Montreal Cognitive Assessment (MoCA)
Version 2

Administration and Scoring Instructions

The Montreal Cognitive Assessment (MoCA) was designed as a rapid screening instrument for mild cognitive dysfunction. It assesses different cognitive domains: attention and concentration, executive functions, memory, language, visuoconstructional skills, conceptual thinking, calculations, and orientation. Time to administer the MoCA is approximately 10 minutes. The total possible score is 30 points; a score of 26 or above is considered normal.

1. Alternating Trail Making:

Administration: The examiner instructs the subject: *"Please draw a line, going from a number to a letter in ascending order. Begin here* [point to (1)] *and draw a line from 1 then to A then to 2 and so on. End here* [point to (E)]."

Scoring: Allocate one point if the subject successfully draws the following pattern:
1 –A- 2- B- 3- C- 4- D- 5- E, without drawing any lines that cross. Any error that is not immediately self-corrected earns a score of 0.

2. Visuoconstructional Skills (Rectangle):

Administration: The examiner gives the following instructions, pointing to the **rectangle**: *"Copy this drawing as accurately as you can, in the space below".*

Scoring: One point is allocated for a correctly executed drawing.

- Drawing must be three-dimensional
- All lines are drawn
- No line is added
- The horizontal lines are relatively parallel.
- The object must be clearly rectangular (i.e., the shorter vertical sides cannot be more than ¾ of the length of the longer horizontal lines.

A point is not assigned if any of the above-criteria are not met.

3. Visuoconstructional Skills (Clock):

Administration: Indicate the right third of the space and give the following instructions: *"Draw a* ***clock****. Put in all the numbers and set the time to 5 past 4".*

Scoring: One point is allocated for each of the following three criteria:
• Contour (1 pt.): the clock face must be a circle with only minor distortion acceptable (e.g., slight imperfection on closing the circle);
• Numbers (1 pt.): all clock numbers must be present with no additional numbers; numbers must be in the correct order and placed in the approximate quadrants on the clock face; Roman numerals are acceptable; numbers can be placed outside the circle contour;
• Hands (1 pt.): there must be two hands jointly indicating the correct time; the hour hand must be clearly shorter than the minute hand; hands must be centred within the clock face with their junction close to the clock centre.

A point is not assigned for a given element if any of the above-criteria are not met.

© Z. Nasreddine MD *www.mocatest.org*

1633 MoCA-Instructions-English Page 1

4. Naming:

Administration: Beginning on the left, point to each figure and say: *"Tell me the name of this animal".*

Scoring: One point each is given for the following responses: (1) giraffe; (2) bear (or specific varieties of bears); (3) hippopotamus (or hippo).

5. Memory:

Administration: The examiner reads a list of 5 words at a rate of one per second, giving the following instructions:
"This is a memory test. I am going to read a list of words that you will have to remember now and later on. Listen carefully. When I am through, tell me as many words as you can remember. It doesn't matter in what order you say them."
Mark a check in the allocated space for each word the subject produces on this first trial. When the subject indicates that (s)he has finished (has recalled all words), or can recall no more words, read the list a second time with the following instructions:
"I am going to read the same list for a second time. Try to remember and tell me as many words as you can, including words you said the first time."
Put a check in the allocated space for each word the subject recalls after the second trial.
At the end of the second trial, inform the subject that (s)he will be asked to recall these words again by saying,
"I will ask you to recall those words again at the end of the test."

Scoring: No points are given for Trials One and Two. Scoring is based on the delayed recall trial.

6. Attention:

Forward Digit Span: Administration: Give the following instruction: "*I am going to say some numbers and when I am through, repeat them to me exactly as I said them*". Read the five number sequence at a rate of one digit per second.

Backward Digit Span: Administration: Give the following instruction: "*Now I am going to say some more numbers, but when I am through you must repeat them to me in the backwards order.*" Read the three number sequence at a rate of one digit per second.

Scoring: Allocate one point for each sequence correctly repeated, (*N.B.*: the correct response for the backwards trial is 2-5-8).

Vigilance: Administration: The examiner reads the list of letters at a rate of one per second, after giving the following instruction: "*I am going to read a sequence of letters. Every time I say the letter A, tap your hand once. If I say a different letter, do not tap your hand*".

Scoring: Give one point if there is zero to one errors (an error is a tap on a wrong letter or a failure to tap on letter A).

© Z. Nasreddine MD

Serial 7s: Administration: The examiner gives the following instruction: *"Now, I will ask you to count by subtracting 7 from 90, and then, keep subtracting 7 from your answer until I tell you to stop."* Give this instruction twice if necessary.

Scoring: This item is scored out of 3 points. Give no (0) points for no correct subtractions, 1 point for one correction subtraction, 2 points for two-to-three correct subtractions, and 3 points if the participant successfully makes four or five correct subtractions. Count each correct subtraction of 7 beginning at 100. Each subtraction is evaluated independently; that is, if the participant responds with an incorrect number but continues to correctly subtract 7 from it, give a point for each correct subtraction. For example, a participant may respond "82 – 75 – 68 – 61 – 54" where the "82" is incorrect, but all subsequent numbers are subtracted correctly. This is one error and the item would be given a score of 3.

7. **Sentence repetition**:

Administration: The examiner gives the following instructions: *"I am going to read you a sentence. Repeat it after me, exactly as I say it* [pause]:
A bird can fly into closed windows when it's dark and windy."

Following the response, say: *"Now I am going to read you another sentence. Repeat it after me, exactly as I say it* [pause]:
The caring grandmother sent groceries over a week ago."

Scoring: Allocate 1 point for each sentence correctly repeated. Repetition must be exact. Be alert for errors that are omissions (e.g., omitting "closed", "over") and substitutions/additions (e.g., "Birds can easily fly into closed windows . . ."; substituting "stormy" for "windy", altering plurals, etc.).

8. **Verbal fluency**:

Administration: The examiner gives the following instruction: *"Tell me as many words as you can think of that begin with a certain letter of the alphabet that I will tell you in a moment. You can say any kind of word you want, except for proper nouns (like Bob or Boston), numbers, or words that begin with the same sound but have a different suffix, for example, love, lover, loving. I will tell you to stop after one minute. Are you ready?* [Pause] *Now, tell me as many words as you can think of that begin with the letter S.* [time for 60 sec]. *Stop."*

Scoring: Allocate one point if the subject generates 11 words or more in 60 sec. Record the subject's response in the bottom or side margins.

9. **Abstraction**:

Administration: The examiner asks the subject to explain what each pair of words has in common, starting with the example: *"Tell me how a carrot and a potato are alike".* If the subject answers in a concrete manner, then say only one additional time: *"Tell me another way in which those items are alike".* If the subject does not give the appropriate response *(vegetable)*, say, *"Yes, and they are also both vegetable".* Do not give any additional instructions or clarification.

© Z. Nasreddine MD

After the practice trial, say: *"Now, tell me how a diamond and a ruby are alike".*
Following the response, administer the second trial, saying: *"Now tell me how a cannon and a rifle are alike".* Do not give any additional instructions or prompts.

Scoring: Only the last two item pairs are scored. Give 1 point to each item pair correctly answered. The following responses are acceptable:
diamond-ruby = gem stones, precious stones, jewels;
cannon-rifle = weapons, guns, used for hurting/killing people, used in war.

The following responses are **not** acceptable:
diamond-ruby = from the earth
cannon-rifle: fires/shoots; ammunition

10. Delayed recall:

Administration: The examiner gives the following instruction: *"I read some words to you earlier, which I asked you to remember. Tell me as many of those words as you can remember."*
Make a check mark (√) for each of the words correctly recalled spontaneously without any cues, in the allocated space.

Scoring: **Allocate 1 point for each word recalled freely without any cues.**

Optional:
Following the delayed free recall trial, prompt the subject with the semantic category cue provided below for any word not recalled. Make a check mark (√) in the allocated space if the subject remembered the word with the help of a category or multiple-choice cue. Prompt all non-recalled words in this manner. If the subject does not recall the word after the category cue, give him/her a multiple choice trial, using the following example instruction, *"Which of the following words do you think it was,* CAR, TRUCK, or PLANE*?"*
Use the following category and/or multiple-choice cues for each word, when appropriate:

TRUCK:	category cue: mode of transportation	multiple choice: car, truck, plane
BANANA:	category cue: type of fruit	multiple choice: pear, apple, banana
VIOLIN:	category cue: type of musical instrument	multiple choice: violin, harp, guitar
DESK:	category cue: type of furniture	multiple choice: chair, desk, bed
GREEN:	category cue: a colour	multiple choice: green, yellow, black

Scoring: **No points are allocated for words recalled with a cue.** A cue is used for clinical information purposes only and can give the test interpreter additional information about the type of memory disorder. For memory deficits due to retrieval failures, performance can be improved with a cue. For memory deficits due to encoding failures, performance does not improve with a cue.

© Z. Nasreddine MD

11. Orientation:

Administration: The examiner gives the following instructions: "Tell me the date today". If the subject does not give a complete answer, then prompt accordingly by saying: *"Tell me the [year, month, exact date, and day of the week]."* Then say: *"Now, tell me the name of this place, and which city it is in."*

Scoring: Give one point for each item correctly answered. The subject must tell the exact date and the exact place (name of hospital, clinic, office). No points are allocated if subject makes an error of one day for the day and date.

TOTAL SCORE: Sum all subscores listed on the right-hand side. Add one point for an individual who has 12 years or fewer of formal education, for a possible maximum of 30 points. A final total score of 26 and above is considered normal.

© Z. Nasreddine MD

NAME :
Education : Date of birth :
Sex : DATE :

MONTREAL COGNITIVE ASSESSMENT (MOCA®)
Version 7.2 Alternative Version

VISUOSPATIAL / EXECUTIVE

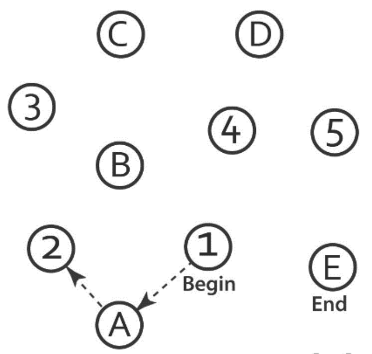

[ ]

Copy rectangle

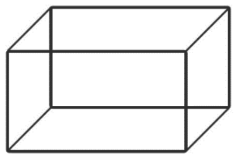

[ ]

Draw CLOCK (Five past four)
(3 points)

[ ] Contour [ ] Numbers [ ] Hands

POINTS ___/5

NAMING

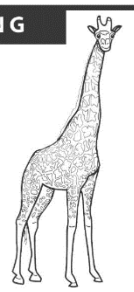

[ ]

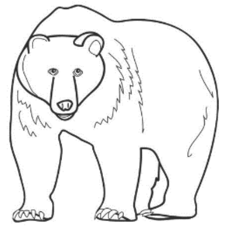

[ ]

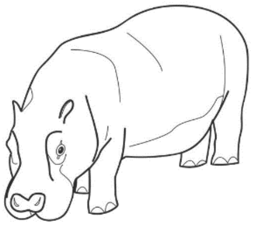

[ ]

___/3

MEMORY

Read list of words, subject must repeat them. Do 2 trials, even if 1st trial is successful. Do a recall after 5 minutes.

	TRUCK	BANANA	VIOLIN	DESK	GREEN
1st trial					
2nd trial					

No points

ATTENTION

Read list of digits (1 digit/ sec.).
Subject has to repeat them in the forward order [ ] 3 2 9 6 5
Subject has to repeat them in the backward order [ ] 8 5 2
___/2

Read list of letters. The subject must tap with his hand at each letter A. No points if ≥ 2 errors
[ ] F B A C M N A A J K L B A F A K D E A A A J A M O F A A B
___/1

Serial 7 subtraction starting at 90 [ ] 83 [ ] 76 [ ] 69 [ ] 62 [ ] 55
4 or 5 correct subtractions: **3 pts**, 2 or 3 correct: **2 pts**, 1 correct: **1 pt**, 0 correct: **0 pt**
___/3

LANGUAGE

Repeat : A bird can fly into closed windows when it's dark and windy. [ ]
The caring grandmother sent groceries over a week ago. [ ]
___/2

Fluency / Name maximum number of words in one minute that begin with the letter S [ ] _____ (N ≥ 11 words)
___/1

ABSTRACTION

Similarity between e.g. carrot - potato = vegetable. [ ] diamond - ruby [ ] cannon - rifle
___/2

DELAYED RECALL

Has to recall words WITH NO CUE	TRUCK [ ]	BANANA [ ]	VIOLIN [ ]	DESK [ ]	GREEN [ ]	Points for UNCUED recall only
**Optional** Category cue						
Multiple choice cue						

___/5

ORIENTATION

[ ] Date [ ] Month [ ] Year [ ] Day [ ] Place [ ] City
___/6

Adapted by : Z. Nasreddine MD, N. Phillips PhD, H. Chertkow MD
© Z.Nasreddine MD www.mocatest.org
Administered by: ______________________

Normal ≥26 / 30
TOTAL ___/30
Add 1 point if ≤ 12 yr edu

MoCA test -English
