# Supplementary material for: Localized Nicardipine Release Implants for Prevention of Vasospasm After Aneurysmal Subarachnoid Hemorrhage: A Randomized Clinical Trial
Source: JAMA Neurol. 2024 Aug 19;81(10):1060–5. doi: 10.1001/jamaneurol.2024.2564 (PMC11334004; doi:10.1001/jamaneurol.2024.2564)
Supplement: Supplement 2. — eMethods 1. Protocol Synopsis eMethods 2. Trial Visit Schedule eResults 1. Patient Disposition eTable 1. Patient Disposition at Visit 7 eResults 2. Adverse Events eTable 2. Adverse Events Overview (Safety Population) eResults 3. Exploratory End Point Analysis eTable 3. Functional Outcome at Week 52 eFigure 1. Length of ICU Stay eFigure 2. Duration of Hospitalization eFigure 3. Maximum Plasma Concentration (cmax) of Nicardipine [file jamaneurol-e242564-s002.pdf]

# Supplemental Online Content

Wessels L, Wolf S, Adage T, et al. Localized nicardipine release implants for prevention of vasospasm after aneurysmal subarachnoid hemorrhage: a randomized clinical trial. *JAMA Neurol*. Published online August 19, 2024. doi:10.1001/jamaneurol.2024.2564

**eMethods 1.** Protocol Synopsis

**eMethods 2.** Trial Visit Schedule

**eResults 1.** Patient Disposition

**eTable 1.** Patient Disposition at Visit 7

**eResults 2.** Adverse Events

**eTable 2.** Adverse Events Overview (Safety Population)

**eResults 3.** Exploratory End Point Analysis

**eTable 3.** Functional Outcome at Week 52

**eFigure 1.** Length of ICU Stay

**eFigure 2.** Duration of Hospitalization

**eFigure 3.** Maximum Plasma Concentration ($c_{max}$) of Nicardipine

This supplemental material has been provided by the authors to give readers additional information about their work.

© 2024 Wessels L et al. *JAMA Neurology*.

## eMethods 1. PROTOCOL SYNOPSIS

| |
|---|
| **TITLE:**<br>**A Phase IIb: Randomised, single-blind, safety, tolerability, efficacy and pharmacokinetic study of NicaPlant® in aneurysmal subarachnoid haemorrhage patients undergoing aneurysm clipping.** |
| **PROTOCOL NO:**<br>BIT-002 |
| **STUDY PHASE:**<br>Phase IIb |
| **INVESTIGATOR STUDY SITES:**<br>This study will be conducted at approximately 6 study sites located in Austria and Germany. |
| **OBJECTIVES:**<br>**Primary Objective**<br>• To assess the efficacy of local nicardipine application via controlled-release polymers (NicaPlant®) on the incidence of moderate to severe cerebral angiographic vasospasm following aneurysmal Subarachnoid Haemorrhage (aSAH).<br>• To assess the safety and tolerability of the selected dose of NicaPlant® (10 implants).<br>**Secondary Objective**<br>• To assess the efficacy of local nicardipine application via controlled-release polymers (NicaPlant®) on the occurrence of:<br>o cerebral infarction following aSAH<br>**Exploratory Objectives**<br>To assess: |

© 2024 Wessels L et al. *JAMA Neurology.*

- The efficacy of local nicardipine application via controlled-release polymers (NicaPlant®) on the occurrence of:
  - early morbidity/mortality following aSAH
- The pharmacokinetic profile of NicaPlant® in plasma and its levels in CSF.
- The effect on clinical outcome following aSAH at week 12.
- The effect on mortality following aSAH at week 12.
- The effect on clinical outcome following aSAH at Week 52.
- The effect on mortality following aSAH at week 52.

**METHODOLOGY:**

This is a Safety, Tolerability, Efficacy and Pharmacokinetic study of NicaPlant® in Subarachnoid Haemorrhage.

This study is a randomised, single-blind, parallel-group design comparing NicaPlant® implants versus standard of care in 40 patients who have had aSAH.

The two groups of patients will be subjected to the following treatments:

- Standard of care and NicaPlant®.
- Standard of care.

Both groups of patients will receive the standard of care according to treatment guidelines [8] and all further medical care will not be different between the groups. Patients in the interventional group will only in addition receive NicaPlant® implants.

NicaPlant® will be administered as per the implantation protocol, (in brief: by placement during microsurgical clipping of the ruptured aneurysm in proximity to all of the exposed cerebral blood vessels).

Patients will be assessed to determine the efficacy of NicaPlant® application to reduce the occurrence of cerebral vasospasm, early morbidity/mortality following aSAH and to prevent cerebral infarction. Cerebral vasospasm will be assessed by analysis of angiograms to determine narrowing of the proximal blood vessels.

Clinical Outcomes will be recorded at 12 weeks and 1 year (week 52) after the aSAH. Ongoing assessments will be made to assess the safety and tolerability of the selected dose of NicaPlant®.

A data safety monitoring board (DSMB) will be set-up to monitor safety throughout the trial period and provide recommendations for any necessary actions. A steering committee will receive and review the blinded reports from the DSMB and take action as appropriate.

© 2024 Wessels L et al. *JAMA Neurology.*

The DSMB will convene after 20 patients have been recruited and monitored till initial hospital discharge or up to day 21, whichever occurs first, for a formal safety review. Recruitment will not be stopped during this time.

The final analysis will be carried out when the last patient completes the week 12 assessment, the database has been locked at this time-point and unblinding has occurred.

Data collected for patients at Week 52 after aSAH will be analysed and included as an addendum to the final report.

**BLINDING**

The study design does not include a placebo implant for ethical reasons; therefore, it is not possible to blind the neurosurgeons performing the aneurysmal clipping procedure to study treatment. Accordingly, the control standard of care control group will receive no intracranial treatment (i.e. no placebo implants).

Patient selection bias is controlled by randomisation; patients will be blinded to which treatment they are receiving (implant plus standard of care or standard of care group) throughout the study i.e. the study is single blinded.

Therefore, the treatment groups are as follows:

- Patients receiving standard of care and NicaPlant® implants

OR

- Patients receiving standard of care

Patients will be assigned a corresponding randomisation code at visit 1; in the operation room, following aneurysm clip ligation, a member of the unblinded operation room team will verify the randomisation code; if the patient is to receive NicaPlant® implants, the implantation procedure goes ahead; if not, no implantation is carried out.

In emergency situations, when the identity of the treatment arm must be made known, the treatment arm of the patient can be seen in the IWRS. Access to the IWRS is held by the responsible neurosurgeon and the pharmacy/ICU-independent personnel preparing the medication.

ICU staff (including those undertaking safety assessments, daily transcranial doppler (TCD), DSA and CT scan readings for patient management and daily Glasgow Coma Scale (GCS) assessments will be blinded to the treatment. Moreover, an independent neuroradiologist will undertake the analysis of the angiograms and computed tomography of the brain for the diagnosis of cerebral vasospasm and new cerebral infarcts for study efficacy endpoint assessment. Trained personnel collecting data on clinical outcome at week 12 and 52 will also be blinded. In cases of DIND or anti-vasospasm rescue therapy as determined by the sites, all required data (e.g. clinical data, case narratives, angiograms, CTs) will be provided to a board of blinded independent experts to adjudicate on the presence of DIND or anti-vasospasm rescue therapy.

© 2024 Wessels L et al. *JAMA Neurology.*

**NUMBER OF PATIENTS:**

Up to 40 patients (20 will receive standard of care, 20 will receive standard of care and NicaPlant®).

Patients that withdraw prior to initial hospital discharge, or day 21, whichever occurs first, will be replaced. Replacement patients will receive the same treatment as the withdrawn patient. A maximum of 10 replacements will be done.

Patients who die will be considered to have completed the study and will not be replaced.

**INCLUSION/EXCLUSION CRITERIA:**

**Inclusion Criteria:**

1. **Austria:** Informed consent is obtained from patients with capacity. If patients lack capacity, informed consent is obtained from a personal legal representative. If no suitable personal legal representative is available and the treatment needs to be given urgently, written informed consent will be obtained as soon as the participant is responsive or a representative is available.

   **Germany:** An independent physician is to be consulted to confirm that the presumed will of the patient on the participation in the study has been determined and respected. Written informed consent will be obtained as soon as the participant regains capacity.
2. Male or female patients aged 18 to 75 years (inclusive).
3. World Federation of Neurological Surgeons (WFNS) grade III-IV.
4. Ruptured saccular aneurysm, confirmed by angiography.
5. Onset of aSAH clinical symptoms within the preceding 48 hours.
6. Treatment of aneurysm via surgical clip ligation within 72 hours of aSAH is achievable.
7. Female patients of child-bearing potential must have a negative pregnancy test (urine or serum) at screening and must agree to use adequate birth control up to 12 weeks after implantation of the study drug. Female patients are considered to be not of child-bearing potential if they have a history of tubal ligation or hysterectomy or are post-menopausal with a minimum of 2 years without a natural menstrual cycle. Male patients must agree to use adequate birth control up to 12 weeks after implantation of the study drug.

**Exclusion criteria:**

1. SAH due to other causes (e.g. trauma, fusiform or mycotic aneurysm).
2. World Federation of Neurological Surgeons (WFNS) grade I, II and V patients.
3. Pregnant or Lactating Women.

© 2024 Wessels L et al. *JAMA Neurology.*

4. Intraventricular or intracerebral blood, in the absence of subarachnoid blood.
5. Treatment of aneurysm via endovascular embolisation.
6. Presence of moderate or severe vasospasm on screening angiography.
7. Any known or CT evidence of previous major cerebral damage.
8. Evidence of a cerebral infarction with neurological deficit on pre-treatment CT
9. History of malignant brain tumours or brain metastasis.
10. Patients who have received an investigational product or participated in another interventional clinical study within 30 days prior to randomisation.
11. Patients with known allergy for Poly(D,L-lactide-co-glycolide) (PLGA) or nicardipine.
12. Major complication during aneurysm repair such as, but not limited to, massive intraoperative haemorrhage, brain swelling, or inability to secure the ruptured aneurysm.

**STUDY INTERVENTIONS/DOSE/ROUTE/REGIMEN:**

NicaPlant® is a nicardipine modified-release formulation, it is presented in the form of a rod-shaped implant (2 mm x 10 mm, 4 mg nicardipine load).

Implantation of NicaPlant®

Following aneurysm clip ligation, 10 NicaPlant® implants will be placed into the basal cisterns, in direct contact with the exposed cerebral blood vessel walls.

The distribution of the implanted polymers will follow a pre-defined protocol dependent on aneurysm location to achieve maximum delivery of nicardipine to the vessel segments at highest risk of vasospasm.

**REFERENCE TREATMENT:**

Standard of care for aSAH patients according to the treatment guidelines. [8]

A main difference to the previous study is the lack of limitations for an additional treatment with Nimodipine or any other treatment according the treatment guidelines since the safety of patient will not be reduced.

**CRITERIA FOR EVALUATION:**

***Safety:***

**Daily assessments:**

- Adverse events (AEs)
- Occurrence of shunt-dependent hydrocephalus (to Day 21±1)
- Occurrence of bacterial meningitis (to Day 21±1)

© 2024 Wessels L et al. *JAMA Neurology.*

- Change in grading of AE severity

**At the defined visits 1 to 5:**

- Vital Signs: blood pressure (BP), pulse rate, respiratory rate and body temperature
- Electrocardiogram (ECG)
- Full blood count, urea and electrolytes, liver function tests, C-reactive protein

Safety assessments will be made from baseline until discharge or day 21±1 and at all subsequent visits.

***Efficacy:***

**Primary efficacy endpoint**

- Incidence of moderate to severe cerebral angiographic vasospasm after aneurysmal subarachnoid haemorrhage, where angiographic vasospasm is defined as a ≥33% reduction in diameter in at least one vessel segment by comparison to preoperative angiography.

**Secondary outcome endpoint**

- Incidence of new cerebral infarcts on CT scan at day 14 ± 1, or before patient discharge (compared with postoperative CT scan).

**Exploratory outcome endpoints**

The exploratory outcome endpoints are:

- Incidence of vasospasm-related morbidity/mortality within 21 days or before patient discharge, defined by at least one of the following:
  a. DIND.
  b. Death caused by vasospasm, delayed ischemic neurological deficit (DIND), infarcts or complications due to anti-vasospasm therapy.
  c. The need for anti-vasospasm rescue therapy.

- Length of Intensive Care Unit (ICU) stay
- Length of Hospital Stay
- Clinical outcome at Week 12 post-aneurysm rupture as measured by:
  a. Modified Rankin Scale (mRS) – “Good Outcome” defined as mRS of 0 to 2
  b. Glasgow Outcome Scale (Extended) (GOSE) – “favourable outcome” of 6-8, “unfavourable outcome” of 0-5
  c. Montreal Cognitive Assessment tool (MoCA) - score 0-30 higher better
  d. Health related quality of life assessed using the 5Q-5D-5L and SF36.

- Long-term Clinical outcome at Week 52 post-aneurysm rupture as measured by:

© 2024 Wessels L et al. *JAMA Neurology*.

a. Modified Rankin Scale (mRS) – "Good Outcome" defined as mRS of 0 to 2
b. Glasgow Outcome Scale (Extended) (GOSE) – "favourable outcome" of 6-8, "unfavourable outcome" of 0-5
c. Montreal Cognitive Assessment tool (MoCA) - score 0-30 higher better
d. Health related quality of life assessed using the EQ-5D-5L and SF36.

*Pharmacokinetics (PK):*
Blood sampling for PK assessment will be carried out in all patients on day of clip ligation (0, 6, 12 and 24 hours post clip ligation, ±20 minutes each), on day 8, day 14 and before discharge or day 21 (whichever occurs first) after aneurysm rupture. Blood sampling for PK assessment will additionally be done every time blood is drawn for medical reasons up to patient discharge or day 21 but only once per day.

Single cerebrospinal fluid (CSF) samples obtained from patients provided for medical reasons of an external ventricular drain (EVD) will be collected every time a CSF sample is removed for medical reasons and up to day patient discharge or to day 21, to determine nicardipine levels.

Only samples collected in patients treated with NicaPlant® will be analysed at the end of this study.

*Pharmacodynamics (PD):*
The PD endpoints are incidence of moderate to severe angiographic measurements of cerebral vasospasm and new cerebral infarcts as assessed by the independent neuroradiologist, and DIND.

**STATISTICAL METHODS:**

*Aim:*

The objective is to evaluate the safety, tolerability and efficacy of 20 patients surgically receiving 10 NicaPlant® implants compared to a group of 20 Control patients receiving standard of care for aSAH. Additionally, the plasma and CSF pharmacokinetics of the 10 Implant group will be evaluated.

*Analysis Sets:*

Three main patient analysis sets will be described: Full Analysis Set (FAS), Per protocol (PP) and Safety sets. The FAS set is considered the primary analysis set for all primary and secondary efficacy endpoints. The PP set will also be used to analyse the primary efficacy endpoint and will consist of those patients in the FAS set without any major protocol deviations. The Safety set is considered the primary set for all safety evaluations.

For the purpose of evaluating PK and PD endpoints, separate Pharmacokinetic and Pharmacodynamic analysis sets will be defined.

*Power and Sample Size:*

© 2024 Wessels L et al. *JAMA Neurology.*

The primary endpoint is the Incidence of Cerebral Angiographic Vasospasm (Moderate to Severe) at Day 8 and these rates are compared for the 10 Implant and Control treatment groups using the Fishers Exact test for differences in proportions.

A total of 40 patients (20 in each group) is in fact sufficient to detect a clinically relevant difference of 50% between the active treatment (10 Implant) and control group (active: 20%, control: 70%) with a two tailed significance level of 5% and 84% power.

*Data Descriptions:*

Data will be summarised by patient population as appropriate, dose group (10 Implant/Control), assessment (visit 1-7 / day where appropriate) and time (where appropriate). Continuous variables will be summarised as descriptive summaries and categorical variables will be summarised as proportions and frequencies. Graphical displays appropriate to these types of data will be used to present important findings.

*Data Analyses:*

The primary endpoint and other binary outcomes (incidence rates) will be evaluated using Fishers exact test.

Ordered categorical variables will be analysed using the Cochran-Mantel-Haenzel (CMH) test (with modified ridits).

Ordinal logistic regression may be supplemented if other covariates of interest are identified.

Normally distributed interval data will be analysed using an analysis of variance (ANOVA) model.

Where appropriate odds ratios or adjusted means together with 95% confidence intervals will be plotted, comparing the two treatment groups (10 Implants/Control).

*Treatment Group Comparability:*

Surgical details (including clip ligation & implantation) and measures at baseline (including: demographics, medical history, concomitant medication, physical examination, pregnancy test) will be summarised descriptively by treatment group (10 Implant/Control) and overall. No formal comparisons of the treatment groups at baseline will be made.

***Safety Endpoints:***

All safety assessments performed including adverse events (and separately shunt-dependent hydrocephalus, bacterial meningitis), vital signs (blood pressure, pulse rate, respiratory rate and body temperature), ECG, haematology and biochemistry will be descriptively summarised. No formal analysis is intended.

***Pharmacokinetics:***

***a. Drug Profiles:***

Plasma - Individual patient plasma drug concentration profiles over time for the active treatment group (10 Implant) will be presented graphically and in summary tables. These will identify assessment day (1 (0, 6, 12 and 24 hours), 8, 14, 21 and additional days if appropriate or CSF sampling days where appropriate).

Overall mean profile summaries for plasma will be produced according to assessment day and time (where appropriate) for the relevant analysis set using descriptive statistics (n, arithmetic mean, SD, min, median, max, geometric mean, and coefficient of variation (CV)) and these will also be presented graphically using geometric means with linear and semi-logarithmic scales.

CSF – Individual patient CSF values will be listed by day, date and time and plotted individually over time as appropriate.

However, these values will only be descriptively and graphically summarised according to the active treatment group (10 Implant) by day/visit if, prior to database lock, there is considered sufficient data to present them in the final statistical and clinical study reports.

***b. PK Parameters:***

Plasma - The individual drug profiles will then be used to derive the respective PK curve parameters for each patient. This will involve non-compartmental methods.

The overall mean PK parameter summaries will then also be produced according to the relevant analysis set, using descriptive statistics (n, arithmetic mean, SD, min, median, max, geometric mean and CV). Plasma PK parameters will be presented for both Day 1 kinetics (0-24 hours) and Day 1 – 21 whenever sufficient data is available. The $AUC_{0-\infty}$, $AUC_{0-t}$ and $C_{max}$ together with other defined parameters for plasma will also be presented graphically where appropriate.

Individual patient plasma PK parameter values will also be presented with the summary tables.

CSF – The $C_{av}$ (Day 1 – 21) for CSF will be similarly presented to the plasma PK parameters if, prior to database lock, there is considered sufficient CSF data to present the $C_{av}$ in the final statistical and clinical study reports.

**STUDY SCHEDULE**

**Study Timings:** Timing in the study is measured from time of aneurysm rupture; as the time of rupture cannot be determined precisely it shall be defined as the time from onset of first symptoms.

| Visit | Time | Procedures |
|---|---|---|

| | | |
|---|---|---|
| Visit 1 | Pre-implantation assessment (within 48 hours of aneurysm rupture) | Consent procedures; inclusion/exclusion criteria; CT scan for aSAH assessment; DSA for vasospasm assessment; demographics/medical history; physical examination; pregnancy test; vital signs; ECG; haematology; serum biochemistry; mGCS; randomisation. |
| Visit 2 | Clip Ligation & Implantation (within 72 hours of aneurysm rupture) | Clip ligation, implantation (if randomised to the study group) of 10 NicaPlant® implants; CT of the brain 36 ± 12 h after aneurysm clipping; PK plasma sampling; CSF sampling (only in EVD patients). |
| Ongoing assessments | Day of surgery to discharge or day 21±1 | Daily: mGCS; TCD reading; recording of occurrence of bacterial meningitis and shunt-dependent hydrocephalus.<br>CSF sampling only in patients provided with EVD and in case the CSF is removed for medical reasons.<br>PK plasma sampling every time blood is drawn for medical reasons but only once per day. |
| Visit 3 | Angiography (day 8±1 post aneurysm rupture and whenever vasospasm is suspected by sonographic or clinical means up to day 8) | Angiographic assessment for vasospasm per DSA; PK plasma sampling. vital signs; ECG; haematology; serum biochemistry |
| Visit 4 | before patient discharge or day 14±1 post aneurysm rupture (whichever occurs first) | CT of the brain for assessment of new cerebral infarcts; PK plasma sampling; vital signs; ECG; haematology; serum biochemistry |
| Visit 5 | before patient discharge or day 21±1 (whichever occurs first). | PK plasma sampling; vital signs; ECG; haematology; serum biochemistry; Modified Rankin Scale (mRS). |
| Visit 6 | Week 12 post aneurysm rupture | Modified Rankin Scale (mRS); extended Glasgow Outcome Scale (GOSE); Montreal Cognitive Assessment tool (MoCA); Health related quality of life assessed using the EQ-5D-5L and SF36; Recording of SAH related mortality. |
| Visit 7 | Week 52 post aneurysm rupture (Unblinded data to be reported in a CSR addendum) | Modified Rankin Scale (mRS); extended Glasgow Outcome Scale (GOSE); Montreal Cognitive Assessment tool (MoCA); Health related quality of life assessed using the EQ-5D-5L and SF36; Recording of SAH related mortality. |

© 2024 Wessels L et al. *JAMA Neurology.*

## eMethods 2 – TRIAL VISIT SCHEDULE

| Visit → | Visit 1 | Visit 2 | Ongoing Assessments | Visit 3 | Visit 4 | Visit 5 | Visit 6 | Visit 7 |
|---|---|---|---|---|---|---|---|---|
| Time → | 0-48hrs After aneurysm rupture | Within 72hrs of aneurysm rupture | Until patient discharge or up to day 21±1 post aneurysm<br>Daily | Day 8±1 after aneurysm rupture | Before patient discharge or day 14 ±1 after aneurysm rupture | Patient discharge or day 21±1 after aneurysm rupture | Week 12±1 after aneurysm rupture | Week 52 ±2 after aneurysm rupture |
| Consent | X | | | | | | | |
| DSA | X | | | X[1] | | | | |
| CT of the brain | X | X[2] | | | X | | | |
| Inclusion/Exclusion | X | | | | | | | |
| Demographics/medical history | X | | | | | | | |
| Physical Examination | X | | | | | | | |
| Pregnancy Test | X | | | | | | | |
| Adverse Event Reporting | X | X | X | | | | | |
| Vital Signs[3] | X | | | X | X | X | | |
| ECG | X | | | X | X | X | | |
| Haematology & Serum biochemistry[4] | X | | | X | X | X | | |

[1] A day 8±1 angiogram will be performed even if the patient has no clinical or sonographic evidence of vasospasm. If the patient develops clinical or sonographic changes suggestive of vasospasm prior to day 8, an angiogram will be performed to confirm vasospasm, this will replace the one scheduled for day 8. If the patients develop clinical or sonographic changes suggestive of vasospasm after day 8 an angiogram will be performed to confirm vasospasm, unless it is clinically not indicated.
[2] 36±12 hours post clip ligation.
[3] Blood pressure, pulse rate, respiratory rate and body temperature.
[4] FBC, U&Es, LFT, CRP.

© 2024 Wessels L et al. *JAMA Neurology.*

| Visit → | Visit 1 | Visit 2 | Ongoing Assessments | Visit 3 | Visit 4 | Visit 5 | Visit 6 | Visit 7 |
|---|---|---|---|---|---|---|---|---|
| Time → | 0-48hrs After aneurysm rupture | Within 72hrs of aneurysm rupture | Until patient discharge or up to day 21±1 post aneurysm | Day 8±1 after aneurysm rupture | Before patient discharge or day 14 ±1 after aneurysm rupture | Patient discharge or day 21±1 after aneurysm rupture | Week 12±1 after aneurysm rupture | Week 52 ±1 after aneurysm rupture |
| Randomisation | X | | | | | | | |
| Clip Ligation & Implantation | | X | | | | | | |
| Post-Surgical Bacterial meningitis | | | X | | | | | |
| Shunt-dependent hydrocephalus | | | X | | | | | |
| PK Plasma[5] | | X[6] | | X | X | X | | |
| CSF Sampling[7] | | X | X | | | | | |
| mGCS for WFNS or DIND assessment | X | | X | | | | | |
| TCD[8] | | | X | | | | | |
| mRS | | | | | | X | X | X |
| GOSE | | | | | | | X | X |
| MoCA | | | | | | | X | X |
| ED-5D-5L | | | | | | | X | X |
| SF36 | | | | | | | X | X |
| Mortality related to SAH | | | | | | X | X | X |

[5] Day 1, 8, 14 and 21, blood collection – a single sample will be collected, the time of sampling has to be recorded. Additional samples will be collected up to day 21 when blood is drawn for medical reasons but only once per day.
[6] Day 1 Plasma samples will be collected at time 0, 6, 12, 24 h post implant, ±20 minutes each .
[7] EVD drain patients only; sample collected when CSF is removed for medical reason up to patient discharge or to day 21.
[8] TCD daily for evidence of vasospasm, if vasospasm is suspected, it needs to be confirmed by DSA.

© 2024 Wessels L et al. *JAMA Neurology.*

## eResults 1. PATIENT DISPOSITION

A total number of 44 subjects were screened and 41 subjects were randomised, treated and accounted for in the Safety population (Implant group: 21 subjects; control group: 20 subjects). There were 3 screening failures.

The primary efficacy analysis involving the Full Analysis Set (FAS) population excluded one subject who was observed post randomisation to have met an exclusion criterion and was therefore withdrawn leaving 20 subjects in each treatment group. The Data Safety and Monitoring Board (DSMB) determined that no subjects should be excluded from the Safety Population and therefore, efficacy results are only presented for the FAS population.

By week 52, a total number of 6 patients had discontinued: 4 from the control group and 2 from the implant group. In the control group, 2 patients had died (1 before week 12 and 1 after week 12), 1 withdrew consent and 1 was lost to follow up at week 52. The Investigator deemed that for the patient who died in the control group prior to week 12 that this was related to SAH. In the implant group, 1 patient who entered the study was subsequently excluded as it was revealed that they had met an exclusion criterion at the time of surgery and 1 patient was lost to follow-up.

**eTable 1.** Patient Disposition at Visit 7 (Week 52±1)

| | | | **Control (n=20)** | **Implant (n=21)** | **Overall (n=41)** |
|---|---|---|---|---|---|
| Screened | | n | | | 44 |
| Randomized | | n (%) | 20 (100.0) | 21 (100.0) | 41 (100.0) |
| Treated | | n (%) | 20 (100.0) | 21 (100.0) | 41 (100.0) |
| Completed | | n (%) | 16 (80.0) | 19 (90.5) | 35 (85.4) |
| Discontinued | | n (%) | 4 (20.0) | 2 (9.5) | 6 (14.6) |
| Reason for discontinuation | *Death* | n (%) | 2 (10.0) | | 2 (4.9) |
| | *Presence of an exclusion criterion* | n (%) | | 1 (4.8) | 1 (2.4) |
| | *Other* | n (%) | 1 (5.0) | 1 (4.8) | 2 (4.9) |
| | *Withdrawal of consent* | n (%) | 1 (5.0) | | 1 (2.4) |
| Was mortality related to SAH | *No* | n (%) | 1 (5.0) | 1 (4.8) | 2 (4.9) |
| | *Yes* | n (%) | 1 (5.0) | | 1 (2.4) |

© 2024 Wessels L et al. *JAMA Neurology.*

## eResults 2. ADVERSE EVENTS

**eTable 2.** Adverse Events Overview (Safety Population)

| Adverse event category | | Control (n=20) | | | Implant (n=21) | | | Overall (n=41) | | |
|---|---|---|---|---|---|---|---|---|---|---|
| | | **n** | **(%)** | **freq** | **n** | **(%)** | **freq** | **n** | **(%)** | **freq** |
| All Adverse Events (AEs) | | 20 | 100.0 | 149 | 21 | 100.0 | 108 | 41 | 100.0 | 257 |
| Pre-Treatment Signs and Symptoms | | 3 | 15.0 | 3 | 0 | 0 | 0 | 3 | 7.3 | 3 |
| Treatment Emergent Adverse Events (TEAEs) | | 20 | 100.0 | 96 | 20 | 95.2 | 84 | 40 | 97.6 | 180 |
| Drug Relationship | *Related* | 5 | 25.0 | 7 | 6 | 28.6 | 9 | 11 | 26.8 | 16 |
| | *Not Related* | 20 | 100.0 | 89 | 20 | 95.2 | 75 | 40 | 97.6 | 164 |
| Intensity | *Mild* | 10 | 50.0 | 15 | 14 | 66.7 | 21 | 24 | 58.5 | 36 |
| | *Moderate* | 17 | 85.0 | 57 | 17 | 81.0 | 48 | 34 | 82.9 | 105 |
| | *Severe* | 12 | 60.0 | 20 | 8 | 38.1 | 15 | 20 | 48.8 | 35 |
| | *Life Threatening* | 4 | 20.0 | 4 | 0 | 0 | 0 | 4 | 9.8 | 4 |
| Serious AEs | | 9 | 45.0 | 10 | 4 | 19.0 | 6 | 13 | 31.7 | 16 |
| AEs Leading to Death | | 1 | 5.0 | 1 | 0 | 0 | 0 | 1 | 2.4 | 1 |
| Post day 21 – Visit 6 | | 9 | 45.0 | 32 | 9 | 42.9 | 14 | 18 | 43.9 | 46 |
| Post Visit 6 | | 2 | 10.0 | 18 | 7 | 33.3 | 10 | 9 | 22.0 | 28 |

Regarding specific TEAEs with high relevance in the context of aSAH, 1/20 patients in the control group suffered bacterial meningitis and 3/20 patients (15%) in the control group versus 3/21 patients (14%) in the implant group developed shunt-dependent hydrocephalus.

© 2024 Wessels L et al. *JAMA Neurology.*

# eResults 3. EXPLORATORY END POINT ANALYSIS

**eTable 3.** Functional Outcome at Week 52

| mRS | Control (n=18)<br>n (%) | Implant (n=19)<br>n (%) | Total (n=37)<br>n (%) |
|---|---|---|---|
| 0 | 3 (16.7) | 6 (31.6) | 9 (24.3) |
| 1 | 6 (33.3) | 3 (15.8) | 9 (24.3) |
| 2 | 3 (16.7) | 7 (36.8) | 10 (27) |
| 3 | 3 (16.7) | 2 (10.5) | 5 (13.5) |
| 4 | 1 (5.6) | 1 (5.3) | 2 (5.4) |
| 5 | 0 (0) | 0 (0) | 0 (0) |
| 6 | 2 (11.1) | 0 (0) | 2 (5.4) |

At 52 weeks, 12/18 patients (66.7%) in the control group and 16/19 patients (84.2%) in the implant group reached favorable outcome. p=0.27 for control vs. implant mRS 0-2 vs. mRS 3-6; Fishers exact test.

© 2024 Wessels L et al. *JAMA Neurology.*

*Length intensive care unit stay and duration of hospitalization*

The mean (95% CI) length of the intensive care unit stay was 30.2 (22.9-37.4) days in the control group and 20.8 (13.9-27.6) days in the implant group (p=0.064) (eFigure 1).

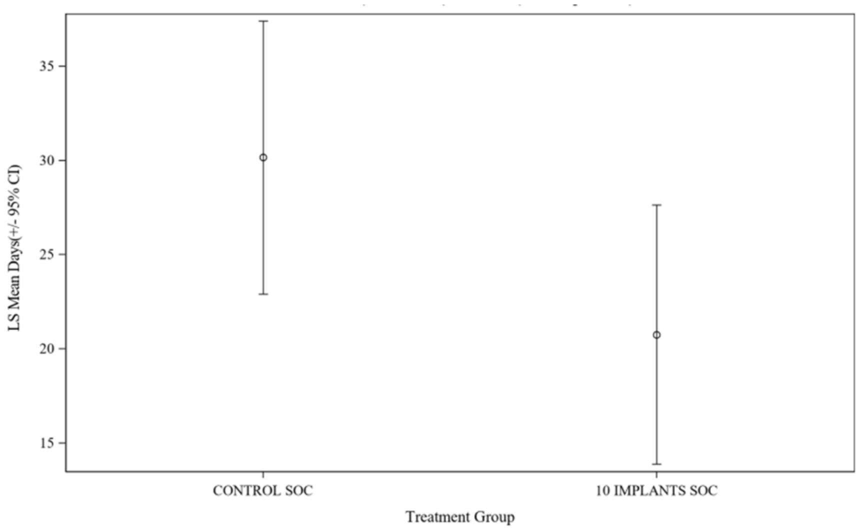


**eFigure 1.** Length of ICU Stay. *Mean length of stay (95 % CI) ANOVA (FAS Population). SOC = Standard of Care*

The mean (95% CI) duration of hospitalization was 45 (27.3-62.7) days in the control group and 29.3 (12.5-46) days in the implant group (p=0.2) (eFigure 2).

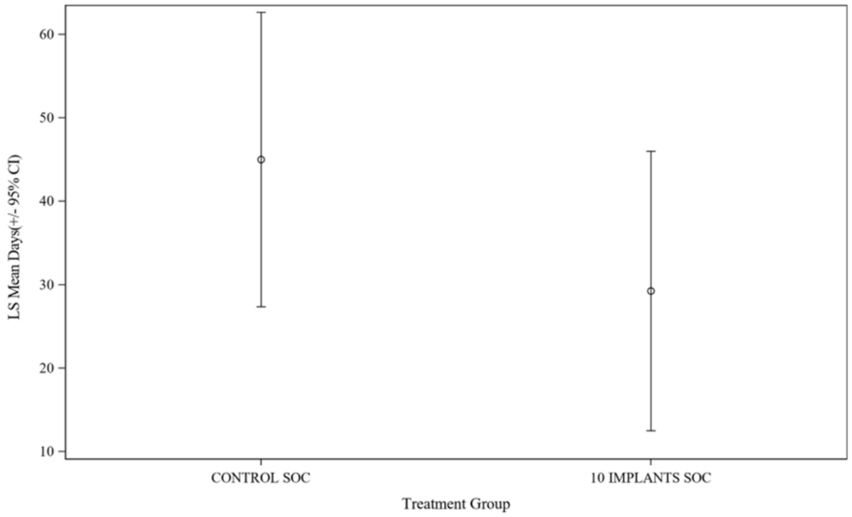


**eFigure 2.** Duration of Hospitalization. *Mean duration (95 % CI) ANOVA (FAS Population). SOC = Standard of Care*

© 2024 Wessels L et al. *JAMA Neurology.*

## Cmax (ng/mL)

| | | 10 IMPLANTS SOC ID | Value |
|---|---|---|---|
| Subject | 1 | 1 | 1.130 |
| | 2 | *4 | *1.77 |
| | 3 | 6 | 1.650 |
| | 4 | 7 | 3.450 |
| | 5 | 8 | 1.270 |
| | 6 | 9 | 1.500 |
| | 7 | 12 | 0.744 |
| | 8 | *13 | *1.48 |
| | 9 | 15 | 1.690 |
| | 10 | 17 | 0.661 |
| | 11 | 20 | 1.720 |
| | 12 | *25 | *0.475 |
| | 13 | *26 | *0.902 |
| | 14 | 29 | 0.953 |
| | 15 | 32 | 0.510 |
| | 16 | *36 | *0.868 |
| | 17 | 38 | 0.821 |
| | 18 | 41 | 1.610 |
| | 19 | 44 | 4.520 |
| Statistic | N | | 19 |
| | MEAN | | 1.459 |
| | SD | | 1.003 |
| | CV | | 68.717 |
| | GM | | 1.228 |
| | MEDIAN | | 1.270 |
| | LQ | | 0.821 |
| | UQ | | 1.690 |
| | MINIMUM | | 0.475 |
| | MAXIMUM | | 4.520 |
| | GSD | | 1.791 |

Footnote: Patient 24 is excluded due to insufficient data
Footnote: *Last sample taken on Day 14 (N.B. Patient 4 last sample day 17)

**eFigure 3.** Maximum Plasma Concentration ($c_{max}$) of Nicardipine. *During the inpatient stay, plasma samples were taken at baseline and at the scheduled visits up to patient discharge or until day 21. Plasma samples for Pharmacokinetic analysis were additionally obtained when blood was drawn for medical reasons but only once per day.*

© 2024 Wessels L et al. *JAMA Neurology.*

*Functional outcome (mRS, GOSE)*

On day 21, clinical functional outcome according to the modified Rankin Scale (mRS) score was assessable in 14/19 patients in the control group (74%) and 13/20 in the implant group (65%). Patients under sedation were excluded from this first assessment. In the control group, 3/14 patients (21.4%) reached favorable outcome (mRS score of 0-2), compared to 5/13 patients (38.5%) in the implant group (p=0.42). At 12-weeks, 10/19 patients (52.6%) in the control group and 12/20 patients (60%) in the implant group presented with favorable outcome (p=0.75). At 52 weeks, 12/18 patients (66.7%) in the control group and 16/19 patients (84.2%) in the implant group reached favorable outcome (p=0.27). Likewise, no difference was detected between the control (16/19 respondents) and implant (19/20 respondents) groups' Glasgow Outcome Scale Extended (GOSE) at week 12 and 52 ($p \geq 0.05$).

*Mortality*

Regarding mortality, only 1 patient died during the first 12 weeks of the study up to week 12 in the control group and there was no statistically significant difference between the two treatment groups ($p \geq 0.05$). At week 52, there were no deaths from the 19 available participants in the implant group and 2 deaths from the 18 available participants in the control group, with no statistically significant difference between the two treatment groups (p=0.23). It should be noted that 1 patient died in implant group but was not included in the Full Analysis Set (FAS) population. This patient was withdrawn from the study after an exclusion criterion was observed post randomisation and clip ligation and died after withdrawal from the study. Due to the exclusion criterion the patient was excluded from the FAS population and only included in the safety population.

*Quality of life and neuropsychological outcome (SF-36, EQ-5D-5L, MoCA)*

Regarding quality of life and neuropsychological outcome at 52 weeks, the SF-36 survey showed no significant difference between groups. Similarly, no difference was noted regarding the EQ-5D-5L (control: 9±3.5, implant: 8.2±4.3; p=0.58) and MoCA test (control: 23.8±2.2, implant: 24.9±3.8; p=0.35). For the SF-36 and EQ-5D-5L at week 52, 19/20 patients of the implant group provided data compared to 14/20 patients of the control group. For the MoCA test at week 52, 17/20 patients of the implant group provided data, compared to 13/20 patients of the control group. Out of the patients that were non-assessable for SF-36, EQ-5D-5L and MoCA, 2 patients of the control group had died, 1 patient of the control group withdrew consent and 1 patient of each group was lost to follow-up. Out of the additional patients that did not complete their SF-36, EQ-5D-5L or MoCA, assessment was hampered due to a severe impairment of consciousness in 2 patients (SF-36, EQ-5D-5L, MoCA), 1 patient was in a poor clinical condition unable to execute the assessment (MoCA) and 1 patient was still admitted to rehabilitation clinic, while her daughter completed the week 52 visit (MoCA). The remaining patient was non-assessable due to a language barrier (MoCA).

© 2024 Wessels L et al. *JAMA Neurology.*
