# Supplementary material for: Localized Nicardipine Release Implants for Prevention of Vasospasm After Aneurysmal Subarachnoid Hemorrhage: A Randomized Clinical Trial
Source: JAMA Neurol. 2024 Aug 19;81(10):1060–5. doi: 10.1001/jamaneurol.2024.2564 (PMC11334004; doi:10.1001/jamaneurol.2024.2564)
Supplement: Supplement 3. — Data Sharing Statement [file jamaneurol-e242564-s003.pdf]

Data Sharing Statement

Wessels. Localized Nicardipine Release Implants for Prevention of Vasospasm After Aneurysmal Subarachnoid Hemorrhage. *JAMA Neurol*. Published August 19, 2024. doi:10.1001/jamaneurol.2024.2564

Data
Data available: No

Additional Information
Explanation for why data not available: The analyzed datasets may not be shared because the patient’s informed consent only permits data analysis and publication by the investigators.
